# Hyperoxygenation revitalizes Alzheimer’s disease pathology through the upregulation of neurotrophic factors

**DOI:** 10.1111/acel.12888

**Published:** 2019-02-11

**Authors:** Juli Choi, Hye‐Jin Kwon, Jung‐Eun Lee, Yunjin Lee, Ju‐Young Seoh, Pyung‐Lim Han

**Affiliations:** ^1^ Department of Brain and Cognitive Sciences Ewha Womans University Seoul Korea; ^2^ Department of Microbiology, College of Medicine Ewha Womans University Seoul Korea; ^3^ Department of Chemistry and Nano Science Ewha Womans University Seoul Korea

**Keywords:** Alzheimer, BDNF, cognition, hyperoxygenation, MeCP2

## Abstract

Alzheimer’s disease (AD) is a neurodegenerative disease characterized by Aβ‐induced pathology and progressive cognitive decline. The incidence of AD is growing globally, yet a prompt and effective remedy is not available. Aging is the greatest risk factor for AD. Brain aging proceeds with reduced vascularization, which can cause low oxygen (O_2_) availability. Accordingly, the question may be raised whether O_2_ availability in the brain affects AD pathology. We found that Tg‐APP/PS1 mice treated with 100% O_2_ at increased atmospheric pressure in a chamber exhibited markedly reduced Aβ accumulation and hippocampal neuritic atrophy, increased hippocampal neurogenesis, and profoundly improved the cognitive deficits on the multiple behavioral test paradigms. Hyperoxygenation treatment increased the expression of BDNF, NT3, and NT4/5 through the upregulation of MeCP2/p‐CREB activity in HT22 cells in vitro and in the hippocampus of mice. In contrast, siRNA‐mediated inhibition of MeCP2 or TrkB neurotrophin receptors in the hippocampal subregion, which suppresses neurotrophin expression and neurotrophin action, respectively, blocked the therapeutic effects of hyperoxygenation on the cognitive impairments of Tg‐APP/PS1 mice. Our results highlight the importance of the O_2_‐related mechanisms in AD pathology, which can be revitalized by hyperoxygenation treatment, and the therapeutic potential of hyperoxygenation for AD.

## 1 INTRODUCTION

Alzheimer's disease (AD), a devastating neurodegenerative disease characterized by Aβ‐induced pathology and progressive cognitive decline, is the most common cause of dementia in elderly individuals (Yankner, Lu, & Loerch, 2008). As the elderly population increases globally, the need for a treatment of AD is rapidly growing, yet a proper method generally applicable to modify disease progression is not still available.

Aging affects virtually all tissues including the brain. Brain aging proceeds with decreases in capillary density, white matter volume, and cognitive function (Ambrose, 2017; Sonntag, Eckman, Ingraham, & Riddle, 2007). Inadequate vascularization can cause a reduction in tissue oxygen (O_2_) availability (Brown & Thore, 2011). When O_2_ availability is low, cells increase expression of hypoxia‐related genes, such as hypoxia‐inducible factor 1α (HIF1α) and vascular endothelial growth factor (VEGF), to cope with metabolic, bioenergetic, and redox demands (Ambrose, 2017). Neurons under sustained hypoxic conditions exhaust homeostatic reserves and undergo adaptive molecular, functional, and structural changes (Sonntag et al., 2007). Animals exposed to hypobaric hypoxia show dendritic atrophy in the hippocampus and cognitive impairment (Titus et al., 2007). Concerning that the incidence rate of AD increases with age (Wyss‐Coray, 2016), the question may be raised whether reduced O_2_ availability in aging brains triggers or aggravates AD pathology. Considering the close relationship between hypoxic conditions and aging‐related changes in the brain, hyperoxygenation therapy might be considered to antagonize hypoxic states and aging‐related changes in the brain of AD patients. However, hyperoxygenation treatment has rarely been studied in research on aging and AD, partly due to the concern that hyperoxygenation may result in harmful oxidative stress (Oter et al., 2005).

Nonetheless, recent studies have reported that hyperoxygenation with 100% O_2_ at 2–3 atmospheres absolute (ATA) is beneficial for treating various brain disorders (Yan, Liang, & Cheng, 2015). Hyperoxygenation treatment improved cognitive sequelae in patients with carbon monoxide poisoning (Weaver et al., 2002) and ameliorated traumatic brain injury (Huang & Obenaus, 2011) and ischemic brain injury (Baynosa et al., 2013). Furthermore, hyperoxygenation treatment (100% O_2_, 2.5 ATA), with 30 intermittent exposures, improved cognitive function in elderly individuals with cognitive deficits (Jacobs, Winter, Alvis, & Small, 1969). Hyperoxygenation treatment (100% O_2_, 2.0 ATA) of poststroke patients for daily 90 min for 40–60 days improved memory impairments on the tasks in the immediate and delayed verbal and nonverbal recall conditions (Boussi‐Gross et al., 2015). Thus, the results of those studies support that hyperoxygenation could produce certain therapeutic effects on neuronal function. However, the mechanism afforded by hyperoxygenation of the brain is poorly understood.

Neurotrophic factors, such as brain‐derived neurotrophic factor (BDNF), neurotrophin 3 (NT3), and neurotrophin 4/5 (NT4/5), play an important role in changes in neuritic morphology and synapse formation through the activation of neurotrophin receptors (Vicario‐Abejón, Owens, McKay, & Segal, 2002) and in neurogenesis (Kang & Schuman, 1995). The levels of BDNF, NT3, and NT4/5 are reduced in the hippocampus of AD patients (Hock, Heese, Hulette, Rosenberg, & Otten, 2000). A decline in the level of BDNF in the forebrain or hippocampus impairs cognitive function (Heldt, Stanek, Chhatwal, & Ressler, 2007). Conversely, administration of BDNF attenuates memory deficits induced by injecting Aβ peptide (Zhang et al., 2015) and a neurotrophin mimetic improves cognitive impairment in AD mice (Prior, Dargusch, Ehren, Chiruta, & Schubert, 2013). Thus, neurotrophic factors are critical for AD pathology.

In the present study, we investigated whether hyperoxygenation treatment changes the Aβ‐induced pathology and cognitive impairment seen in Tg‐APP/PS1 mice. Our analyses demonstrated that hyperoxygenation treatment improved the Aβ pathology and cognitive deficits of Tg‐APP/PS1 mice through the induction of MeCP2‐mediated neurotrophin expression.

## 2 RESULTS

### 2.1 Hyperoxygenation suppressed the expression of hypoxia‐related markers in hippocampal neurons

HT22 hippocampal cells treated with Aβ42 showed increased expression of hypoxia‐related genes, including hypoxia‐inducible factor‐1α (*Hif‐1α*), *Vegf‐a, Hmox1*, and *Pdk1*. In contrast, treatment of HT22 cells with perfluorodecalin (PFD), a synthetic biomaterial that noncovalently dissolves large amounts of molecular oxygen (O_2_) (Lowe, Davey, & Power, 1998), reversed the Aβ42‐induced increase in these markers (Supporting information Figure S1a–d). We examined whether similar changes could occur in the AD‐like brain. Tg‐APP/PS1 mice, which express high levels of Aβ in the brain at 8 months of age (Kim et al., 2012), showed increased expression of hypoxia‐related markers, including *Hif‐1α*, *Hmox1*, and *Pdk2*, in the hippocampus, whereas Tg‐APP/PS1 mice treated with hyperoxygenation (HO_2_; 100% O_2_, 2 ATA) for 1 hr daily for 28 days had reduced expression of these factors (Supporting information Figure S1e–f).

### 2.2 Hyperoxygenation upregulated the expression of neurotrophins in the hippocampus

**Figure 1:**
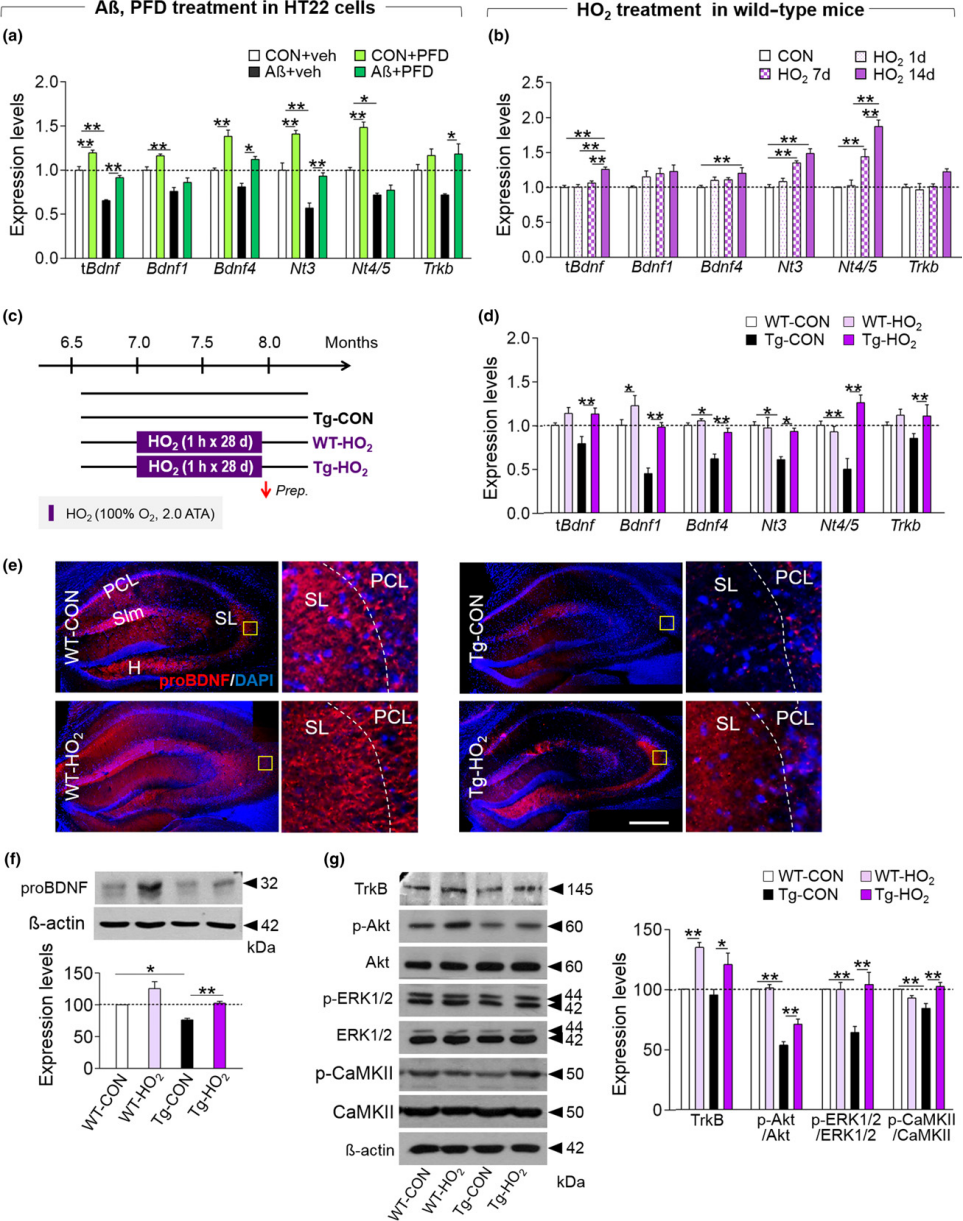
HO_2_ treatment upregulated the expression of neurotrophic factors in the hippocampus of Tg‐APP/PS1 mice. (a) Expression levels of total *Bdnf* (t*Bdnf*), *Bdnf1*, *Bdnf4*, *Nt3*, *Nt4/5*, and *Trkb* in HT22 cells treated with Aβ42 (25 μM) or Aβ42 plus PFD (20%). (b) Expression levels of t*Bdnf*, *Bdnf1*, *Bdnf4*, *Nt3*, *Nt4/5*, and *Trkb* in the hippocampus of young normal mice treated with HO_2_ (100% O_2_, 2 ATA) for 1 hr daily for 1, 7, or 14 days. (c and d) Experimental design (c). Mice were treated with HO_2_ (100% O_2_, 2 ATA) from 7.0 months of age for 1 hr daily for 28 days. Arrow, time point for tissue preparation. Expression levels of t*Bdnf*, *Bdnf1*, *Bdnf4*, *Nt3*, *Nt4/5*, and *Trkb* in the hippocampus of WT‐CON, WT‐HO_2_, Tg‐CON, and Tg‐HO_2_ mice (d). (e) Photomicrographs showing proBDNF expression in the hippocampus of WT‐CON, WT‐HO_2_, Tg‐CON, and Tg‐HO_2_. High magnification (right panels) of the boxed area. PCL, pyramidal cell layer; SL, stratum lucidum; Slm, stratum lacunosum‐moleculare; H, hilus. Red, proBDNF; Blue, DAPI. Scale bar, 500 μm. (f and g) Western blots showing expression levels of proBDNF (f), TrkB, p‐Akt, Akt, p‐ERK1/2, ERK1/2, p‐CaMKII, and CaMKIIα (g) in the hippocampus of WT‐CON, WT‐HO_2_, Tg‐CON, and Tg‐HO_2_ and their quantification levels. Data are presented as mean ± SEM. *p < 0.05; **p < 0.01 (one‐way ANOVA followed by Newman–Keuls *post hoc* test and two‐way ANOVA followed by Bonferroni *post hoc* test)

HT22 cells treated with Aβ42 showed reduced expression of *Bdnf*, *Nt3*, and *Nt4/5*, and *Trkb,* whereas PFD treatment reversed the decrease in the expression of these factors (Figure 1a). The expression of *Bdnf*, *Nt3*, and *Nt4/5 *was also increased in the hippocampus of wild‐type mice that were exposed to HO_2_ (100% O_2_, 2 ATA) for 1 hr daily for more than 7 days (Figure 1b), suggesting that the expression of neurotrophic factors is regulated by hyperoxygenation in vitro and in vivo.

Tg‐APP/PS1 mice had reduced expression of *Bdnf*, *Nt3*, and *Nt4/5*, and *Trkb* in the hippocampus, whereas Tg‐APP/PS1 mice treated with HO_2_ showed increased expression of these factors (Figure 1c,d). Western blotting and immunohistochemical analyses indicated that Tg‐APP/PS1 mice had reduced expression of proBDNF in the hippocampus, whereas HO_2_ treatment reversed the decreased expression of this factor (Figure 1e,f). The levels of TrkB (a common receptor for BDNF, NT3, and NT4/5) and its key signaling mediators, p‐Akt, p‐ERK1/2, and p‐CaMKII, were decreased in the hippocampus of Tg‐APP/PS1 mice, whereas HO_2_ treatment reversed the decreased expression of those factors (Figure 1g).

HO_2_ treatment in Tg‐APP/PS1 mice also tended to increase the expression of the myelination markers *Mobp* and *Mog *in the hippocampus (Supporting information Figure S1a,b). Tg‐APP/PS1 mice had reduced expression of the dendritic marker MAP2 in the hippocampus, whereas HO_2_ treatment in these mice notably upregulated the expression of this marker (Supporting information Figure S2c–e). Tg‐APP/PS1 mice had reduced levels of the neurogenesis markers DCX and Ki‐67 in the dentate gyrus, whereas HO_2_ treatment reversed the reduction of those markers (Supporting information Figure S2f–i).

### 2.3 HO_2_ treatment suppressed the Aβ accumulation in the brain of Tg‐APP/PS1 mice

**Figure 2:**
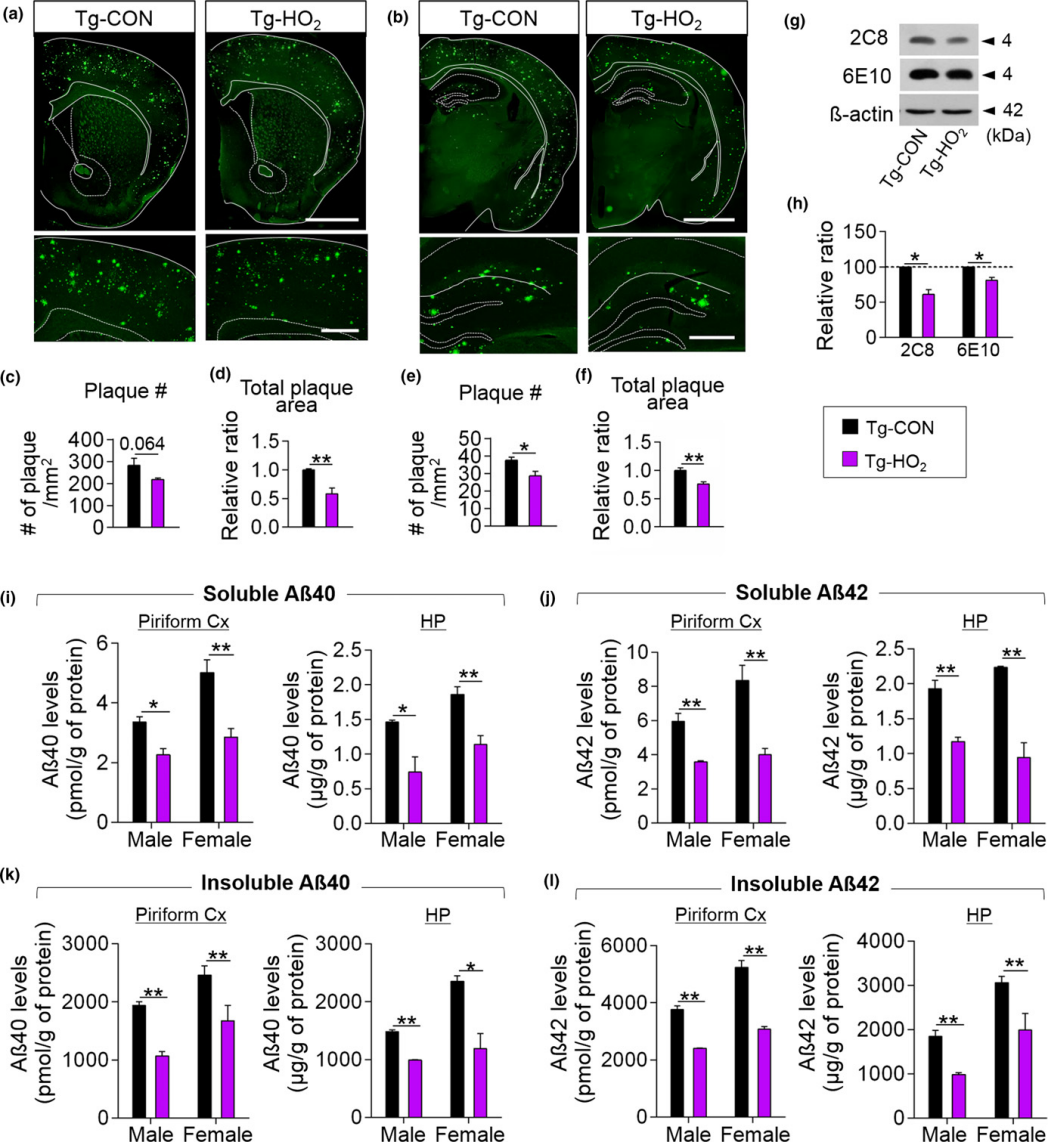
HO_2_ treatment suppressed the Aβ accumulation in the brain of Tg‐APP/PS1 mice. (a–f) Photomicrographs showing thioflavin S‐stained coronal sections at the level of the striatum (a) and hippocampus (b) of brains from Tg‐CON and Tg‐HO_2_ mice. Higher magnification of the prefrontal cortex (a, low panels) and hippocampus (b, low panels). Quantification of the number of plaques (c,e) and the size of stained plaque area (d,f) in the prefrontal cortex (c,d) and hippocampus (e,f). The color codes for Tg‐CON and Tg‐HO_2_ mice in the figure legends are applicable to all parts of Figure 2. Scale bars in upper panels, 2 mm; in lower panels, 500 μm. (g and h) Western blots showing Aβ levels (g) in the hippocampus of Tg‐CON and Tg‐HO_2_ mice. Quantification levels (h). 2C8 and 6E10, Aβ‐specific monoclonal antibodies. (i–l) ELISA data showing Tris‐soluble (i and j) and Tris‐insoluble (k and l) Aβ(40) and Aβ(42) levels in the piriform cortex (Pfcx) and hippocampus (HP) of Tg‐CON and Tg‐HO_2_ mice. Data are presented as mean ± SEM. *p < 0.05; **p < 0.01 (Student's t test)

Next, we examined whether HO_2_ treatment changed Aβ pathology. Thioflavin S (ThS) staining indicated that HO_2_ treatment in Tg‐APP/PS1 mice reduced the overall number and total stained area of ThS‐positive plaques in the prefrontal cortex and hippocampus compared to those of untreated Tg‐APP/PS1 mice (Figure 2a–f). Western blot analysis confirmed a reduction of Aβ level after HO_2_ treatment (Figure 2g,h). Tg‐APP/PS1 mice treated with HO_2_ had reduced levels of the soluble and insoluble forms of Aβ40 and Aβ42 in the piriform cortex and hippocampus (Figure 2i‐l). Overall, these results suggest that HO_2_ treatment reduced Aβ accumulation in Tg‐APP/PS1 mice.

### 2.4 HO_2_ treatment partially suppressed the ROS levels accumulated in the brain of Tg‐APP/PS1 mice

Oxidative stress is elevated by amyloidopathy in AD (Dumont & Beal, 2001). We examined whether or not HO_2_ treatment in mice increased ROS levels in the brain. Analyses of ROS levels by staining with the superoxide sensitive dye dihydroethidium (DHE), immunological staining for 4‐hydroxynonenal (HNE), a lipid peroxidation marker, or the biochemical assessment of the level of malondialdehyde (MDA), another lipid peroxidation marker, indicated that Tg‐APP/PS1 mice had increased ROS levels in the hippocampus, whereas those increases were significantly suppressed after HO_2_ treatment (Supporting information Figure S3a–h). The expression levels of *Cox1*, *eNos*, *nNos*, *Ho‐1*, and the NADPH oxidase subunits in the hippocampus tended to increase in Tg‐APP/PS1 mice, whereas HO_2_ treatment suppressed those increases. Among antioxidant genes, the level of *Prx3* increased after HO_2_ treatment (Supporting information Figure S3i–k). Overall, these results suggest that hyperoxygenation partially reduced, rather than increased, oxidative stress levels in the hippocampus of Tg‐APP/PS1 mice.

### 2.5 Hyperoxygenation‐induced neurotrophin expression was mediated by MeCP2/p‐CREB

**Figure 3:**
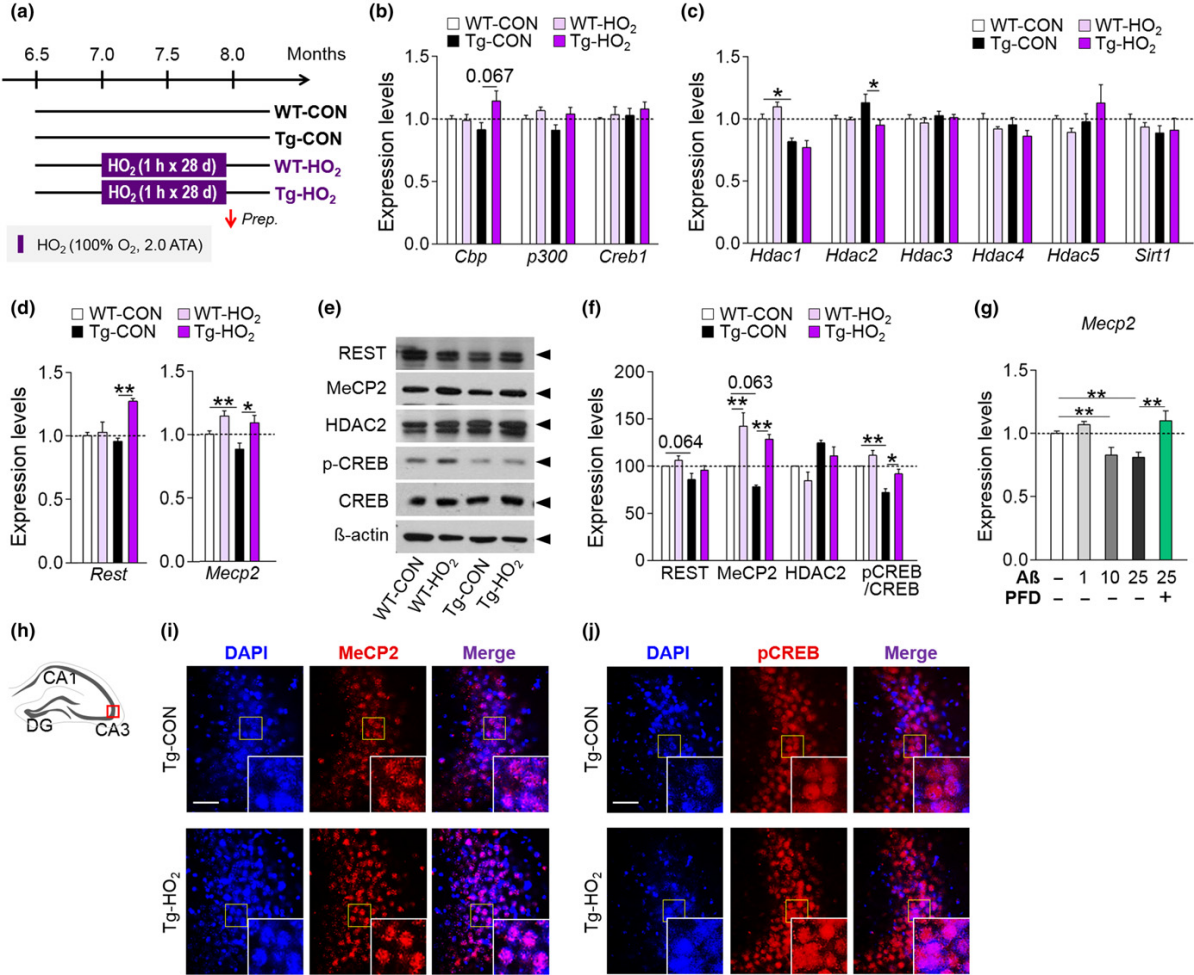
HO_2_ increased MeCP2 and p‐CREB expression in the hippocampus of Tg‐APP/PS1 mice. (a) Experimental design. Mice were treated with HO_2_ (100% O_2_, 2 ATA) from 7 months of age for 1 hr daily for 28 days. Arrow, time point for tissue preparation. (b–d) Real‐time PCR data showing expression levels of *Cbp*, *p300*, *Creb* (b), *Hdac1*, *Hdac2*, *Hdac3*, *Hdac4*, *Hdac5*, *Sirt1* (c), *Rest*, and *Mecp2* (d) in the hippocampus of WT‐CON, WT‐HO_2_, Tg‐CON, and Tg‐HO_2_ mice. (e and f) Western blots showing expression levels of REST1, MeCP2, HDAC2, and p‐CREB (e) in the hippocampus of WT‐CON, WT‐HO_2_, Tg‐CON, and Tg‐HO_2_ mice. Quantification levels (f). (g) Real‐time PCR data showing expression levels of MeCP2 in HT22 cells treated with Aβ (25 μM) or Aβ(25 μM)plus PFD (20%). (h–j) Photomicrographs showing MeCP2 (i) and p‐CREB (j) expression in the CA3 region (h) of WT‐CON, WT‐HO_2_, Tg‐CON, and Tg‐HO_2_ mice. Insets, high magnification of the boxed areas. MeCP2 or p‐CREB, red; DAPI, blue. Scale bars, 50 μm. Data are presented as mean ± SEM. *p < 0.05; **p < 0.01 (one‐way ANOVA followed by Newman–Keuls *post hoc* test and two‐way ANOVA followed by Bonferroni *post hoc* test)

Previous studies have reported that BDNF expression can be regulated by cAMP response element binding protein (CREB) (Koo et al., 2015), histone deacetylase 2 (HDAC2) (Guan et al., 2009), Methyl‐CpG binding protein 2 (MeCP2) (Chang, Khare, Dani, Nelson, & Jaenisch, 2006), or repressor element‐1 transcription factor 1 (REST1) (Goldberg & Coulter, 2014). Therefore, we examined whether HO_2_ treatment in mice changed the expression of these factors. Real‐time PCR and western blot analysis indicated that Tg‐APP/PS1 mice had a reduced expression of MeCP2 and p‐CREB in the hippocampus, whereas HO_2_ treatment reversed the decrease in those factors (Figure 3a–f). The REST1 and HDAC2 protein levels in Tg‐APP/PS1 mice were not significantly changed by HO_2_ treatment (Figure 3e,f). HT22 cells treated with Aβ42 had reduced level of *Mecp2*, whereas PFD treatment reversed the decreased expression of *Mecp2* (Figure 3g). Immunohistological analyses indicated that MeCP2, p‐CREB, and HDAC2 were heavily expressed in the pyramidal and granule cell layers of the hippocampus (Figure 3h–j and Supporting information Figure S4).

Next, we examined whether MeCP2 and p‐CREB play a role in hyperoxygenation‐induced neurotrophin expression. The siRNA‐mediated inhibition of MeCP2 in HT22 cells suppressed the expression of *Bdnf4*, *Nt3*, and *Nt4/5*, and CREB and p‐CREB levels (Supporting information Figure S5a–c). HT22 cells treated with PFD showed increased expression of *Mecp2*, *Bdnf4*, *Nt3*, and *Nt4/5* transcripts relative to control cells, whereas siRNA‐mediated inhibition of MeCP2 suppressed PFD‐induced increase in the levels of *Bdnf4*, *Nt3*, and *Nt4/5* transcripts and of p‐CREB (Supporting information Figure S5d–g). The siRNA‐mediated inhibition of Creb1 also similarly but slightly differently reduced the expression of *Bdnf1*, *Bdnf4*, *Nt3*, and *Nt4/5* (Supporting information Figure S5h,i), and reduced CREB, p‐CREB, and MeCP2 (Supporting information Figure S5j). The siRNA‐mediated inhibition of Creb1 in HT22 cells also suppressed PFD‐induced increased expression of *Mecp2*, *Bdnf4*, *Nt3*, and *Nt4/5* (Supporting information Figure S5k).

### 2.6 HO_2_ treatment increased MeCP2/p‐CREB binding to the promoter of neurotrophin genes

**Figure 4:**
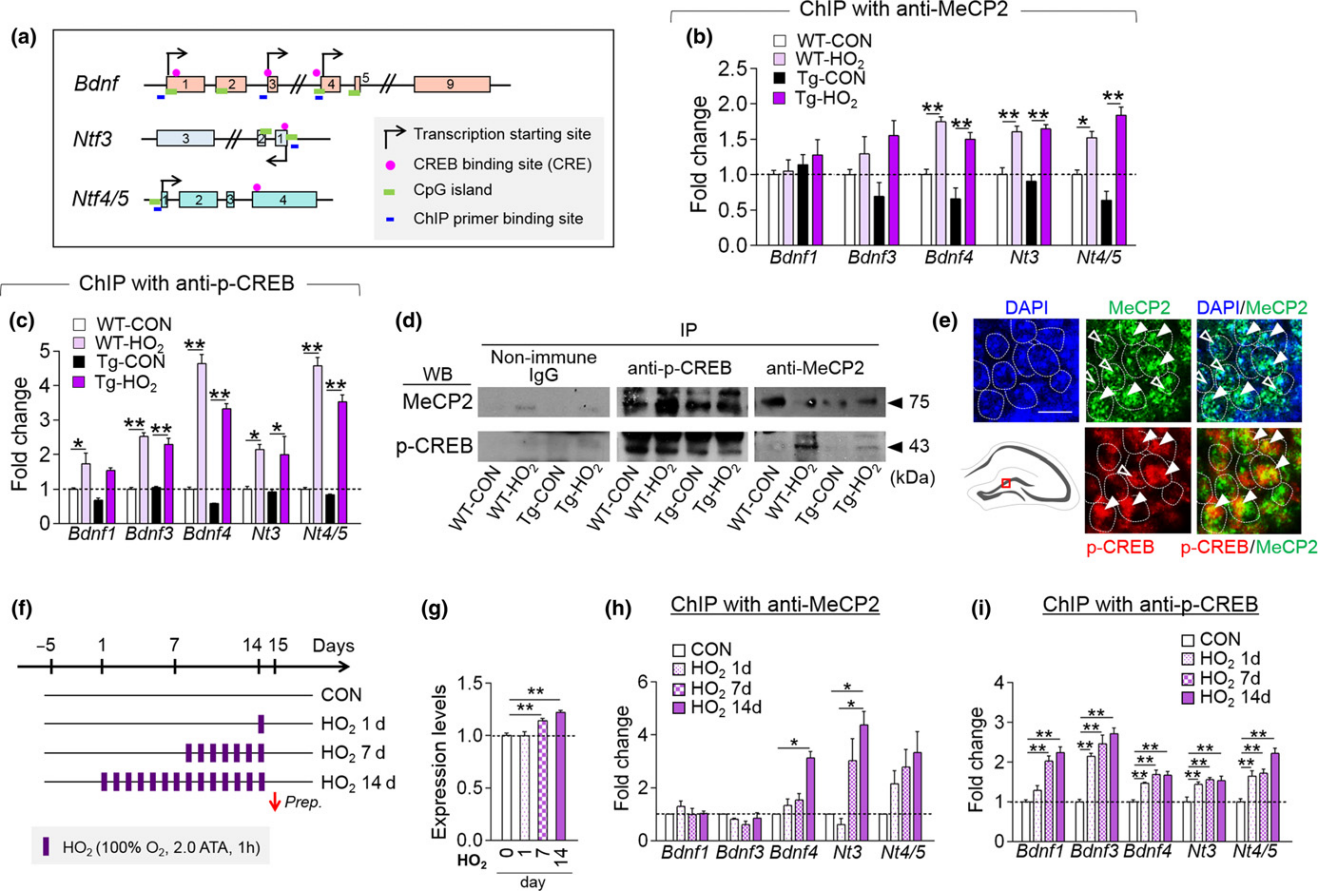
HO_2_ increased MeCP2 and p‐CREB binding to the promoter of neurotrophin genes. (a) Maps of the promoter region of *Bdnf*, *Nt3*, and *Nt4/5* genes. Exons (box), transcription start sites, CRE, CpG islands, and ChIP PCR‐primer locations are indicated. (b,c) ChIP assays showing MeCP2 (b) and p‐CREB (c) binding to the promoter of *Bdnf1*, *Bdnf3*, *Bdnf4*, *Nt3*, *Nt4/5* in the hippocampus of WT‐CON, WT‐HO_2_, Tg‐CON, and Tg‐HO_2_. (d) Western blots showing coimmunoprecipitation of MeCP2 and p‐CREB from the hippocampal tissue of WT‐CON, WT‐HO_2_, Tg‐CON, and Tg‐HO_2_. (e) Immunofluorescent images showing the colocalization of MeCP2 and p‐CREB in granule neurons of DG subregion (box in the diagram). MeCP2, green; p‐CREB, red; DAPI, blue. Scale bar, 20 μm. (f–i) Experimental design. Mice were treated with HO_2_ (100% O_2_, 2 ATA) for 1 hr daily for 1, 7, or 14 days (f). MeCP2 levels (g) and ChIP assay data showing MeCP2 (h) and p‐CREB (i) binding levels to the promoter of *Bdnf1*, *Bdnf3*, *Bdnf4*, *Nt3*, *Nt4/5* in the hippocampus of mice treated with HO_2_ for indicated days. Data are presented as mean ± SEM. *p < 0.05; **p < 0.01 (one‐way ANOVA followed by Newman–Keuls *post hoc* test and two‐way ANOVA followed by Bonferroni *post hoc* test)

Next, we examined whether HO_2_ treatment increased neurotrophin expression via MeCP2/p‐CREB activity in Tg‐APP/PS1 mice. ChIP assays indicated that HO_2_ treatment in Tg‐APP/PS1 mice increased the levels of MeCP2 binding to the promoter of *Bdnf4*, *Nt3*, and *Nt4/5*, but not *Bdnf1*, and of p‐CREB binding to the promoter of *Bdnf3*, *Bdnf4*, *Nt3*, and *Nt4/5* (Figure 4a–c). Similar to those in HT22 cells, MeCP2 and p‐CREB were coimmunoprecipitated from hippocampal tissue, and coprecipitant levels increased after HO_2_ treatment (Figure 4d). Immunofluorescence staining indicated that MeCP2 and p‐CREB were colocalized at the single cell level in pyramidal neurons and in granule neurons in the hippocampus (Figure 4e). MeCP2 expression in the hippocampus increased when mice were treated with HO_2_ for 7 day or more (Figure 4f,g). ChIP assay data supported these findings. Both MeCP2 and p‐CREB binding to the promoter of *Bdnf*, *Nt3*, and *Nt4/5* increased in a manner proportional to the number of HO_2_ treatment days, and their HO_2_‐induced increases became significant when HO_2_ was given for 7 days or more. Interestingly, p‐CREB binding to the promoter of *Bdnf3/4*, *Nt3*, and *Nt4/5* was increased even by a single HO_2_ exposure (Figure 4h,i).

HT22 cells treated with PFD had increased expression and nuclear colocalization levels of MeCP2 and p‐CREB (Supporting information Figure S5l,m). MeCP2 and p‐CREB were co‐immunoprecipitated in HT22 cells, and their coprecipitant levels increased after treatment with PFD (Supporting information Figure S5n). A chromatin immunoprecipitation (ChIP) assay indicated that the binding of MeCP2 and p‐CREB to the proximal promoter of BDNF4, NT3, and NT4/5 markedly increased after PFD treatment in HT22 cells (Supporting information Figure S5o,p).

### 2.7 HO_2_ treatment improved the cognitive deficits of Tg‐APP/PS1 mice

**Figure 5:**
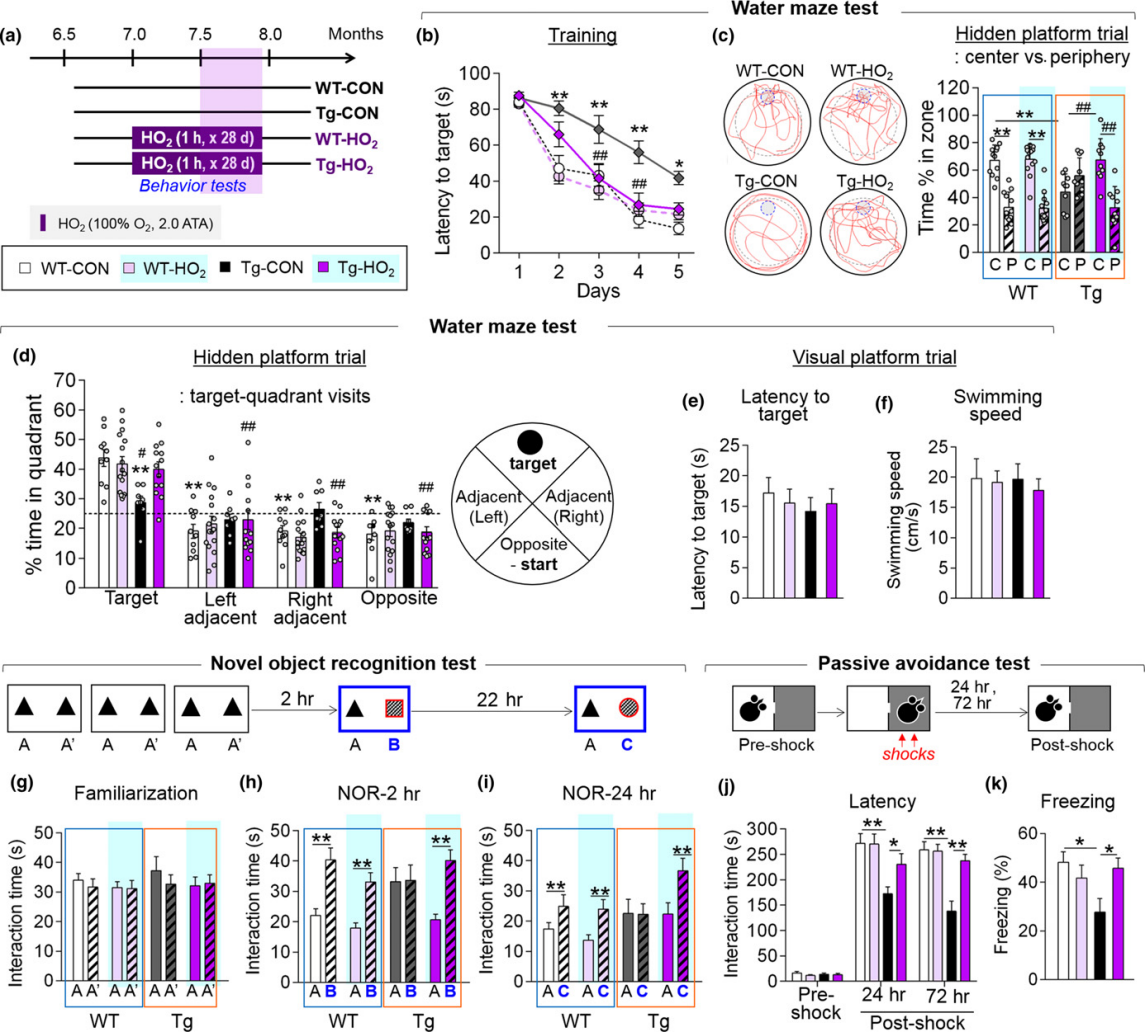
HO_2_ improved the cognitive deficits of Tg‐APP/PS1 mice. (a) Experimental design. Mice were treated with HO_2_ (100% O_2_, 2 ATA) from 7 months of age for 1 hr daily for 28 days. Behavioral tests were performed in this order: water maze test (WM), novel object recognition test (NOR), and passive avoidance test (PA). The color codes in the legends are applicable to all parts of Figure 5. (b) The latency to finding the hidden platform during the training in the water maze test for WT‐CON, WT‐HO_2_, Tg‐CON, and Tg‐HO_2_. (c and d) Representative tracking in the hidden platform trial. The percent time spent in the periphery vs. the center (c) and in each quadrant. Representative tracking of each group (d). Dashed line on (d) indicates a 25% chance of finding the platform. C, center; P, periphery; T, target; L, left; R, right; O, opposite. (e and f) The latency to finding the platform (e) and swim speed (f) during the visual platform trial of WT‐CON, WT‐HO_2_, Tg‐CON, and Tg‐HO_2_. (g–i) The effects of HO_2_ on novel object recognition memory. Time spent exploring between the two identical objects during the familiarization (g), and between a new and an old object 2 hr after familiarization (h, NOR‐2h) and 24 hr after familiarization (i, NOR‐24h). (j and k) The effects of HO_2_ on fear memory in the PA. The latency to entering the dark chamber at the preshock, and 24 and 72 hr after shock (j), and the freezing time 24 hr after shock (k). Data are presented as mean ± SEM. WT‐CON, n = 7–18; WT‐HO_2_, n = 8–18; Tg‐CON, n = 7–12; Tg‐HO_2_, n = 8–14 per each group. *p < 0.05; **p < 0.01, difference between indicated groups; ^#^ *p *< 0.05; ^##^ *p *< 0.01, difference between Tg‐CON and Tg‐HO_2_ (two‐way ANOVA followed by Bonferroni *post hoc* test and two‐way repeated‐measures ANOVA followed by Bonferroni *post hoc* test)

Next, we examined whether HO_2_ treatment changed behaviors. To address this, Tg‐APP/PS1 mice treated with HO_2_ (100% O_2_, 2 ATA), starting from 7 months of age, for 1 hr daily for 2 weeks were placed in the indicated behavioral tests while continuing HO_2_ treatment on the given schedule (Figure 5a). Tg‐APP/PS1 mice at 7.5 months of age display severe cognitive deficits (Kim et al., 2012). During the training phase of the water maze test, Tg‐APP/PS1 mice given HO_2_ exhibited a markedly reduced latency to find the hidden platform compared to Tg‐APP/PS1 control mice (Figure 5b). On the following probe trial, Tg‐APP/PS1 mice with HO_2_ treatment spent less time exploring the periphery of the pool and also more time exploring the target quadrant compared to Tg‐APP/PS1 control mice (Figure 5c,d). During the visual platform trial, both the latency to reaching the visual platform and swimming speed were comparable among all test groups (Figure 5e,f). In the novel object recognition memory test, Tg‐APP/PS1 mice did not show a preference for a novel object over a familiar one, whereas Tg‐APP/PS1 mice given HO_2_ treatment showed a preference for a novel object over a familiar one when examined 2 or 24 hr after training (Figure 5g–i). In the passive avoidance test, Tg‐APP/PS1 mice showed a reduced latency to entering the shock‐associated dark chamber, along with reduced freezing in the light chamber, whereas HO_2_ treatment in Tg‐APP/PS1 mice reversed these behavioral changes as examined 24 or 72 hr after shock (Figure 5j,k).

However, Tg‐APP/PS1 mice exposed to moderate hyperoxygenation (mHO_2_; 42% O_2_, 2 ATA), starting from 7 months of age, for 1 hr daily for 2 weeks or more, in a manner similar to the treatment with 100% O_2_ at 2 ATA (Supporting information Figure S6a), showed the same cognitive deficits in the water maze test (Supporting information Figure S6b–f) and novel object recognition test (Supporting information Figure S6g–i), as the Tg‐APP/PS1 control mice. In the passive avoidance test, Tg‐APP/PS1 mice given mHO_2_ treatment exhibited an increased latency to enter the dark chamber 24 hr after foot shock and a tendency toward increased latency 72 and 120 hr after shock (Supporting information Figure S6j).

**Figure 6:**
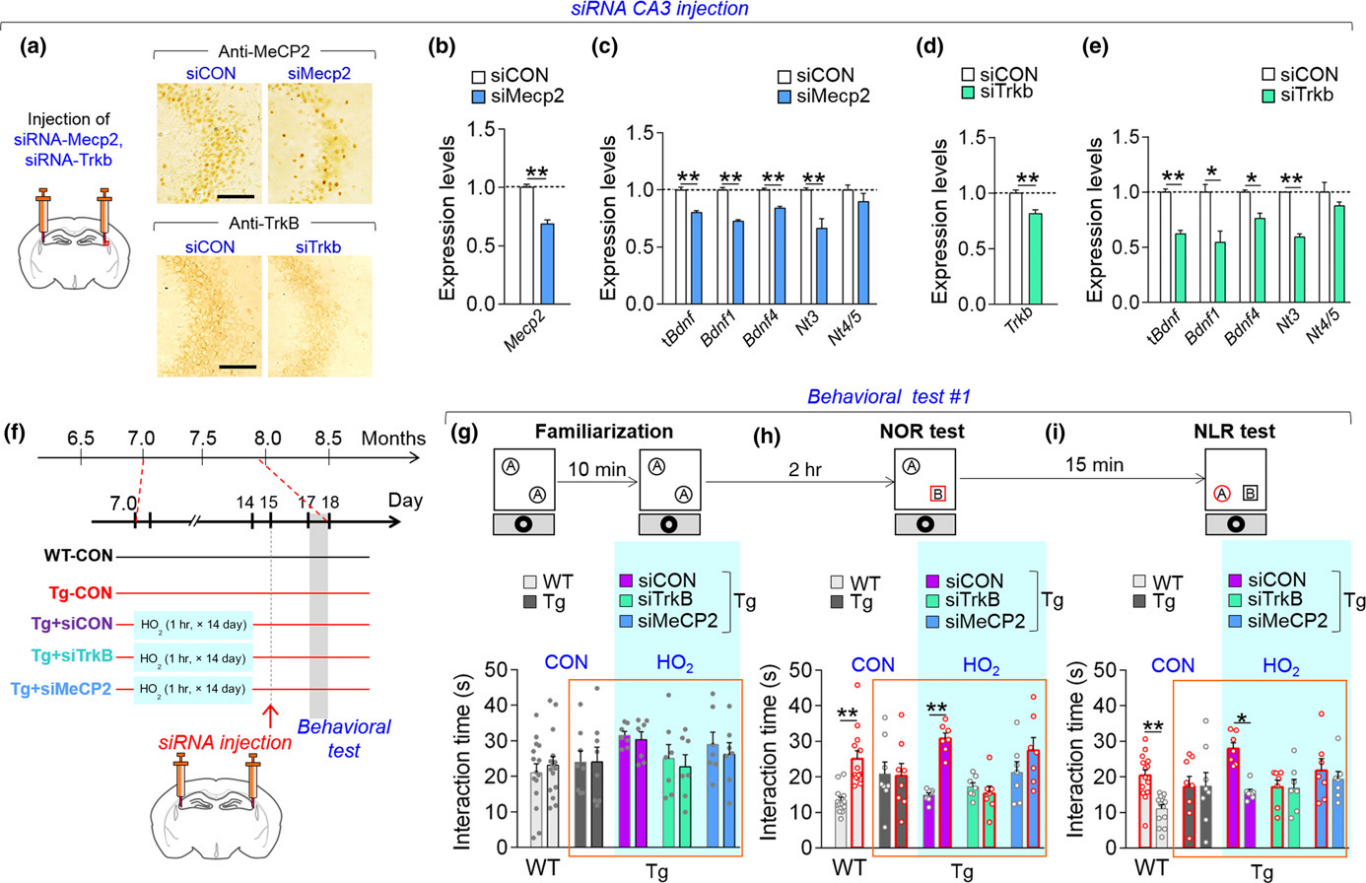
Inhibition of *Mecp2* or *Trkb* in the CA3 subregion blocked the therapeutic effects of HO_2_. (a–e) Photomicrographs showing siRNA‐mediated knockdown of MeCP2 and TrkB in the CA3 region (a). Expression levels of t*Bdnf*, *Bdnf1*, *Bdnf4*, *Nt3*, *Nt4/5*, *Mecp2* (b) and *Trkb* (d) in the CA3 of mice injected with siRNA‐MeCP2 (b,c) or siRNA‐TrkB (d,e). Scale bar, 20 μm. (f) Experimental design. Mice were treated with HO_2_ (100% O_2_, 2 ATA) from 7 months of age for 1 hr daily for 14 days. The siRNA‐MeCP2 or siRNA‐TrkB was injected into the CA3 subregion of the hippocampus on day 15, and behavioral tests were performed in all mice on day 17. WT‐CON, wild‐type control; Tg‐CON, Tg‐APP/PS1 mice; Tg‐siCON, Tg‐siMeCP2; and Tg‐siTrkB, Tg‐APP/PS1 mice treated with HO_2_ followed by injection with siRNA‐control, siRNA‐MeCP2, or siRNA‐TrkB, respectively. (g–i) Time spent exploring the two identical objects during the familiarization (g), between a new and an old object 2 hr after familiarization (h, NOR), and between a displaced object and an old object from the previous test (i, NLR). The black circle is a spatial marker posted outside the open field. Blue shaded, mouse groups treated with HO_2_. Data are presented as mean ± SEM. WT‐CON +siCON, n = 13; Tg‐CON +siCON, n = 8; Tg‐HO_2_ + siCON, n = 7; Tg‐HO_2_ + siTrkB, n = 7; Tg‐HO_2_ + siMeCP2, n = 7 per each group. *p < 0.05; **p < 0.01 (Student's t test and two‐way ANOVA followed by Bonferroni *post hoc* test)

### 2.8 Inhibition of MeCP2 or TrkB in the CA3 subregion blocked the therapeutic effects of HO_2_ on object recognition memory

Next, we investigated whether the therapeutic effects of HO_2 _were mediated by the neurotrophin genes and their actions in the hippocampus. Given that multiple neurotrophin genes were induced by HO_2_ treatment (Figure 1) and that MeCP2 was required for HO_2_‐induced neurotrophin expression (Figure 3), we investigated whether inhibition of *Trkb*, a common receptor for *Bdnf*, *Nt3*, and *Nt4/5*, or independently inhibition of *Mecp2*, within the hippocampus could block the behavioral effects of HO_2_.

We confirmed that the siRNA‐mediated inhibition of *Mecp2* in the CA3 of wild‐type animals reduced *tBdnf*, *Bdnf1*, *Bdnf4*, *Nt3*, *Creb1*, and *Trkb* expression (Figure 6a–e). The siRNA‐mediated inhibition of *Trkb* in the CA3 also suppressed the levels of *tBdnf*, *Bdnf1*, *Bdnf4*, and *Nt3* (Figure 6a–e).

Tg‐APP/PS1 mice treated with HO_2_ (100% O_2_, 2 ATA) from 7 months of age for 14 days, followed by injection of siRNA‐MeCP2 or siRNA‐TrkB into the CA3 subregion (Figure 6f), did not distinguish between novel and familiar objects in the novel object recognition (NOR) test (Figure 6g,h). In the subsequent novel location recognition (NLR) test, Tg‐APP/PS1 mice injected with siRNA‐TrkB or siRNA‐MeCP2 also did not preferentially explore the displaced object relative to the non‐moved object, despite the fact that they received HO_2_ treatment (Figure 6i). The total interaction time with the objects in these tests was comparable among the test groups.

## 3 DISCUSSION

We demonstrated that HO_2_ treatment suppressed the Aβ accumulation (Figure 2) and hippocampal neuritic atrophy (Supporting information Figure S2) and improved the cognitive deficits in Tg‐APP/PS1 mice (Figures 5 and 6). These results support the notion that O_2_ availability is critical for AD‐like pathology. A postmortem study indicated that 77% of vascular dementia patients had AD pathology (Barker et al., 2002). Higher cerebral blood flow velocity, measured by a transcranial Doppler flowmetry, was correlated with a lower prevalence of cognitive decline and dementia (Ruitenberg et al., 2005). Patients diagnosed with amnestic mild cognitive impairment (MCI) aged 65 years and above advance to AD at a rate of 15% per year (Davatzikos, Bhatt, Shaw, Batmanghelich, & Trojanowski, 2011). A recent study reported that subjects with MCI showed global brain hypoperfusion and low oxygen metabolism in the brain (Liu et al., 2014). Together, these studies raise the possibility that the cellular and molecular mechanisms, which might be hypoxia‐related mechanisms as inferred from the results reversed by hyperoxygenation, are involved in AD‐related pathology. Further studies to elaborate the physiological significance of this possibility are highly encouraged.

HO_2_ (100% O_2, _2.0 ATA) treatment profoundly improved the cognitive deficits of Tg‐APP/PS1 mice (Figures 5 and 6), whereas moderate HO_2_ (42% O_2_, 2.0 ATA) treatment on the same schedule did not (Supporting information Figure S6). HO_2_ treatment for more than 7 days in wild‐type mice was required for the increase in MeCP2 expression (Figure 4f–i). These results suggest that the repeated and sufficient HO_2_ stimulation is required for the therapeutic effects of HO_2_. In normal physiological conditions, normal O_2_ concentration in arterial blood in humans is 9.5%, whereas the partial O_2_ concentration in the brain drops to 3.4% (McKeown, 2014). HO_2_ treatment with 100% O_2_ at 2.0 ATA increases the O_2_ tension in the brain tissues by 7–10‐fold compared to the pO_2_ normally achieved with room air at 1.0 ATA (Demchenko et al., 2005). In the presence of high O_2_, the sulfur‐containing amino acids cysteine and methionine can be reversibly oxidized (Sanchez, Riddle, Woo, & Momand, 2008). Cysteine and methionine oxidations are known to play a role in antioxidant defense, redox sensing, and regulation, and changes in protein activity (Kim et al., 2014). Therefore, it is possible that HO_2_‐initiated oxygenation of unidentified proteins might play a role in HO_2_‐induced changes.

Hyperoxygenation is known to cause harmful oxidative stress (Oter et al., 2005). However, it was also suggested that certain therapeutic effects of hyperoxygenation act through oxidative stress brought about by hyperoxia (Thom, 2009). A recent study with a rat model of stroke reported the protective effect of repeated HO_2_ treatment (100% O_2, _1.3 ATA, 45 min/once, 40 sessions) was blocked by inhibition of ROS (Hu et al., 2014). It remains to be explored whether or not the initial and/or mildly increased oxidative stress during each HO_2_ exposure is required for the ultimately therapeutic effects of HO_2_. The ROS levels, as measured by DHE staining, anti‐HNE staining, and MDA assay, in the brain of Tg‐APP/PS1 mice treated with hyperoxygenation tended to be higher than those of age‐matched wild‐type control (Supporting information Figure S3). Thus, our results indicated that the repeated HO_2_ treatment conditions partially suppressed the oxidative stress in the brain of Tg‐APP/PS1 mice (Supporting information Figure S3).

The results of present study support that HO_2_ treatment procedures can be developed for people with AD. However, HO_2_ treatment at certain conditions cloud produce some complications such as middle ear, lung, or sinus barotrauma when hyperbaric pressure is used, and neurological, retinal, and pulmonary oxygen toxicity caused by high oxygen level (Grim, Gottlieb, Boddie, & Batson, 1990; Leung & Lam, 2018). Pulmonary toxicity occurs when exposed to HO_2_ at over 2.5 ATA for 6 hr or 1.5 ATA for over 12 hr at one session (Clark et al., 1999). HO_2_ treatment at 2.0 ATA or higher conditions can develop a risk of seizure (Jain, Torbati, Tao, & Ni, 1999), although its incidence is low (Yildiz, Aktas, Cimsit, Ay, & Toğrol, 2004). Therefore, possible side effects and complications of HO_2_ treatment should be studied in conjunction with specific conditions of AD or other disease states. It also requires to investigate possible side effects of prolonged treatments with HO_2_.

Neurotrophic factors play a role in synaptic plasticity, neuronal circuit activity, and circuit formation (Vicario‐Abejón et al., 2002) and have neuroprotective roles also in hypoxic‐ischemic brain injury (Huang et al., 2017). BDNF expression can be regulated by multiple factors, including CREB (Koo et al., 2015) and MeCP2 (Chang et al., 2006). MeCP2 overexpression in mouse cortical neurons increased the level of BDNF (Klein et al., 2007), and exogenous BDNF revived defective synaptic transmission in MeCP2 null mice (Kline, Ogier, Kunze, & Katz, 2010). In our study, HO_2_ treatment increased the expression of BDNF, NT‐3, and NT‐4/5 through the upregulation of MeCP2/p‐CREB activity (Figures 2 and 3), whereas siRNA‐mediated knockdown of MeCP2 or TrkB in the hippocampus blocked the therapeutic effects of HO_2_ on the cognitive deficits of Tg‐APP/PS1 mice (Figure 6). Thus, our results suggest that HO_2_‐induced MeCP2 upregulation triggers not only BDNF, but also NT‐3, and NT‐4/5 in the hippocampus. Moreover, increased these neurotrophic factors appear to act through TrkB receptors in the hippocampus. Our results highlight the importance of the mechanisms revived by hyperoxygenation in AD pathology, which have not been carefully explored in AD research.

## 4 EXPERIMENTAL PROCEDURES

The detailed procedures and information are described in the Supporting information Appendix S1 and Table S1.

### 4.1 Animals

Tg‐APPswe/PS1dE9 (Tg‐APP/PS1) mice, which show plaque deposition from 6.5 months of age and severe cognitive deficits at 7–7.5 months of age (Kim et al., 2012), were used. All animals were handled in accordance with the animal care guidelines of the Ewha Womans University (IACUC 16‐019).

### 4.2 Hyperoxygenation treatment in mice

Mice were treated with hyperoxygenation (HO_2_) using a hyperbaric chamber as described previously (Kim et al., 2014). Mice were exposed to 100% oxygen daily for 60 min at 2.0 ATA in a hyperbaric chamber for the indicated number of days. For moderate hyperoxygenation (mHO_2_), mice were treated as above, but with atmospheric air.

### 4.3 ELISA assay of Aβ accumulation

ELISA assessments of Aβ(1–40) and Aβ(1–42) levels were carried out as described previously (Kim et al., 2012).

### 4.4 Chromatin immunoprecipitation assay

Chromatin immunoprecipitation (ChIP) assays were performed as described previously (Kim, Lee, Kim, et al., 2016). The chromatins were sheared to 200–800 bp sizes using an EpiShear Probe Sonicator (Active Motif, Carlsbad, CA, USA). For immunoprecipitation, 10 μg of sheared chromatin was added to 2 μg of primary antibody, and 20 μl of protein G magnetic beads was used. The antibodies used were anti‐MeCP2 (3456S; Cell Signaling), antiphospho‐CREB (06‐519; Millipore), and nonimmune rabbit IgG (ab37415; Abcam). Immunoprecipitated DNA was used for quantitative real‐time PCR, as described above.

### 4.5 Stereotaxic injection of siRNA

Stereotaxic injection of siRNA was performed as described previously (Choi et al., 2015). In brief, diluted siRNA (50 ng/μl) was mixed with Neurofect transfection reagent (T800075; Genlantis, San Diego, CA, USA). The siRNA mix (1.8 μl of 7.5 ng/μl) was injected into each CA3 (stereotaxic coordinate: AP, −1.9; ML, ±3.0; DV, −2.1 mm) using a stereotaxic injection system (Vernier Stereotaxic Instrument, Leica Biosystems, Wetzlar, Germany).

### 4.6 Behavioral tests

Behavioral tests were carried out as described previously (Choi et al., 2015). Behavioral performance was recorded with a video‐tracking system (SMART; Panlab S.I., Barcelona, Spain) and a webcam recording system (HD Webcam C210, Logitech, Newark, CA, USA).

### 4.7 Water maze test

The water maze test was performed as described previously (Kim et al, 2012). The training was performed in a circular tank pool filled with opaque water (made using Sargent^®^ White Art Tempera Paint) twice a day for 5 days. On day 6, mice were given the probe trial test. At the end of the experiment, mice were placed on the visible platform test to control for possible locomotor and visual deficits.

#### 4.7.1 Novel object recognition tests

The standard and modified novel objective recognition tests were performed as described previously (Kim, Lee, Park, et al., 2016). A subject mouse was presented to two identical objects (object A) for 10 min. Two hours after the familiarization sessions, one familiar object was replaced with a new object (object B). The time spent exploring each object was recorded for 10 min. Twenty‐four hours, the subject mouse was presented to a familiar object (object A) and a third, new object (object C), and the time spent with each object was recorded for 10 min and analyzed in a blind manner with two researchers.

#### 4.7.2 Modified novel object recognition test and novel location recognition test

A spatial cue was provided on top of the wall directly outside of the open‐field apparatus by posting a black circle marker. A subject mouse was familiarized with two identical objects (object A) for 10 min. Two hours later, the subject mouse was placed in the open field in which the object close to the black circle marker was replaced with a new object (object B). The amount of time spent exploring each object was recorded for 10 min (NOR test). After 15 min of the NOR test, the subject mouse was presented in the open field in which the old familiar object A was moved to a novel location toward the black circle and 20 cm away from object B. The amount of time spent exploring each object was recorded for 10 min (NLR test) and analyzed in a blind manner with two researchers.

#### 4.7.3 Passive avoidance test

The passive avoidance test has been described previously (Kim et al, 2012). The test apparatus consisted of a lighted chamber (1,500 lx) and a dark chamber equipped with a metal grid floor. On the first day, subject mice were individually placed in the lighted chamber with the door opened and allowed to explore freely the equipment for 5 min. On the second day, mice were given with two foot shocks. On the test day, the latency to entering the dark chamber was recorded. The total freezing time during the testing period was manually analyzed.

### 4.8 Statistical analysis

Two‐sample comparisons were carried out using Student's t test, whereas multiple comparisons were performed using one‐way ANOVA followed by the Newman–Keuls post hoc test or two‐way ANOVA or two‐way repeated‐measures ANOVA followed by the Bonferroni post hoc test. All data are presented as mean ± SEM, and statistical significance was accepted at the 5% level.

## AUTHORS’ CONTRIBUTION

JYS and HPL developed the concept and initial experiments; JC and HPL designed the detailed experiments; JC, HJK, JEL, and YL carried out experiments; JC and HPL analyzed and interpreted collected data, and wrote the manuscript; all authors contributed feedback and edited the manuscript.
