# Supplementary figures and images for: Hyperoxygenation revitalizes Alzheimer’s disease pathology through the upregulation of neurotrophic factors
Source: Aging Cell. 2019 Feb 11;18(2):e12888. doi: 10.1111/acel.12888 (PMC6413661; doi:10.1111/acel.12888)

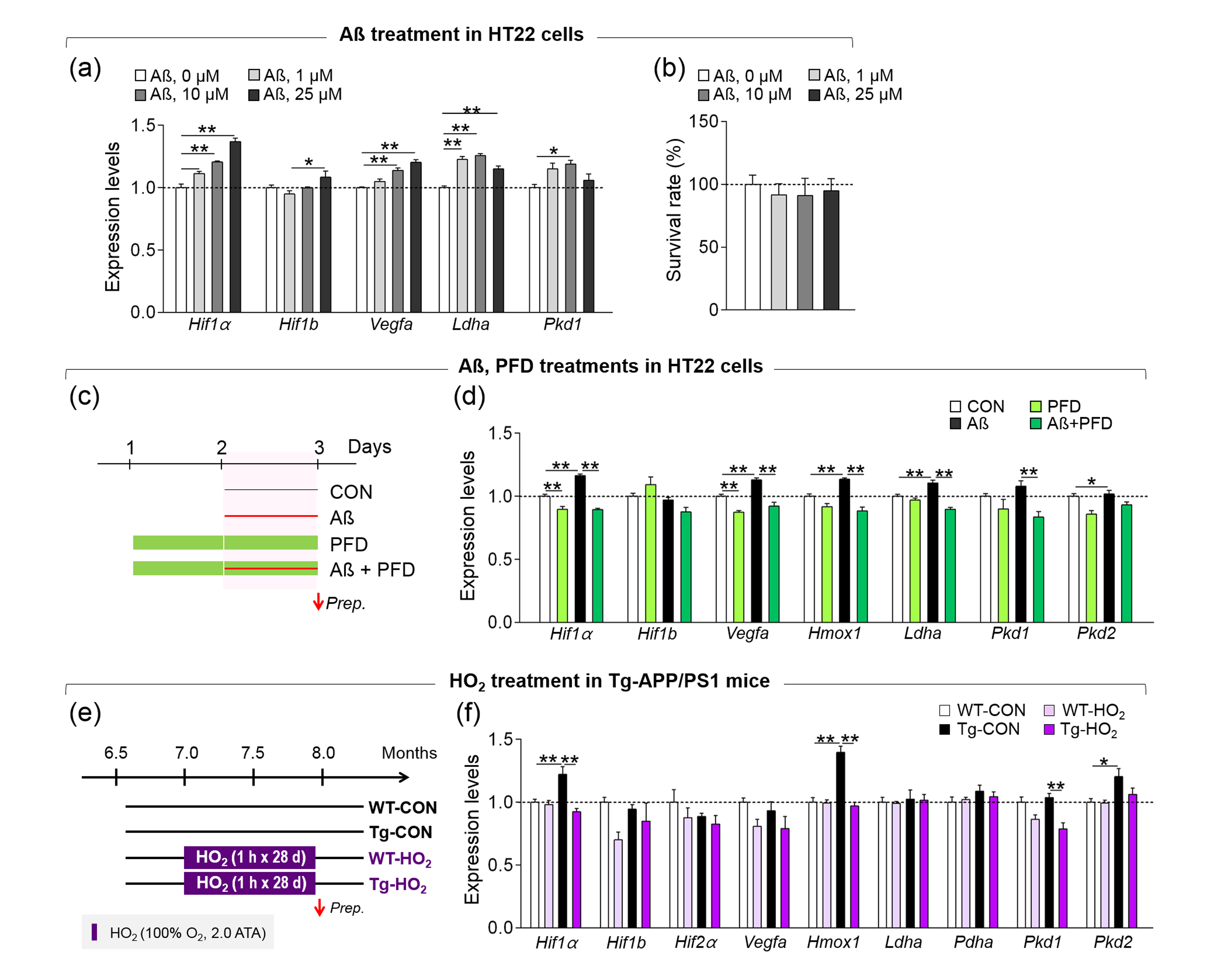

Supplement: Supplementary file 1 [file ACEL-18-e12888-s001.tif]

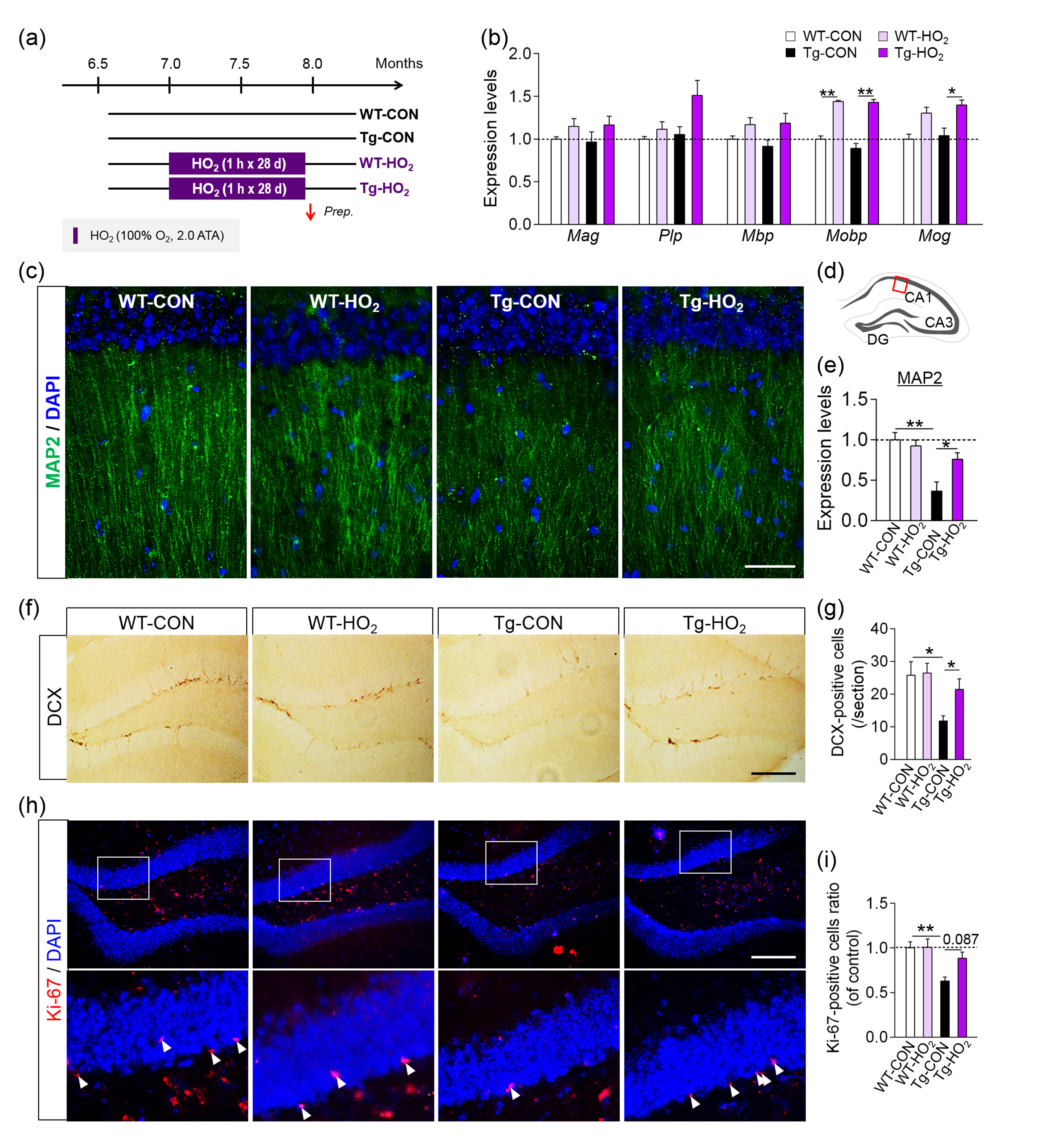

Supplement: Supplementary file 2 [file ACEL-18-e12888-s002.tif]

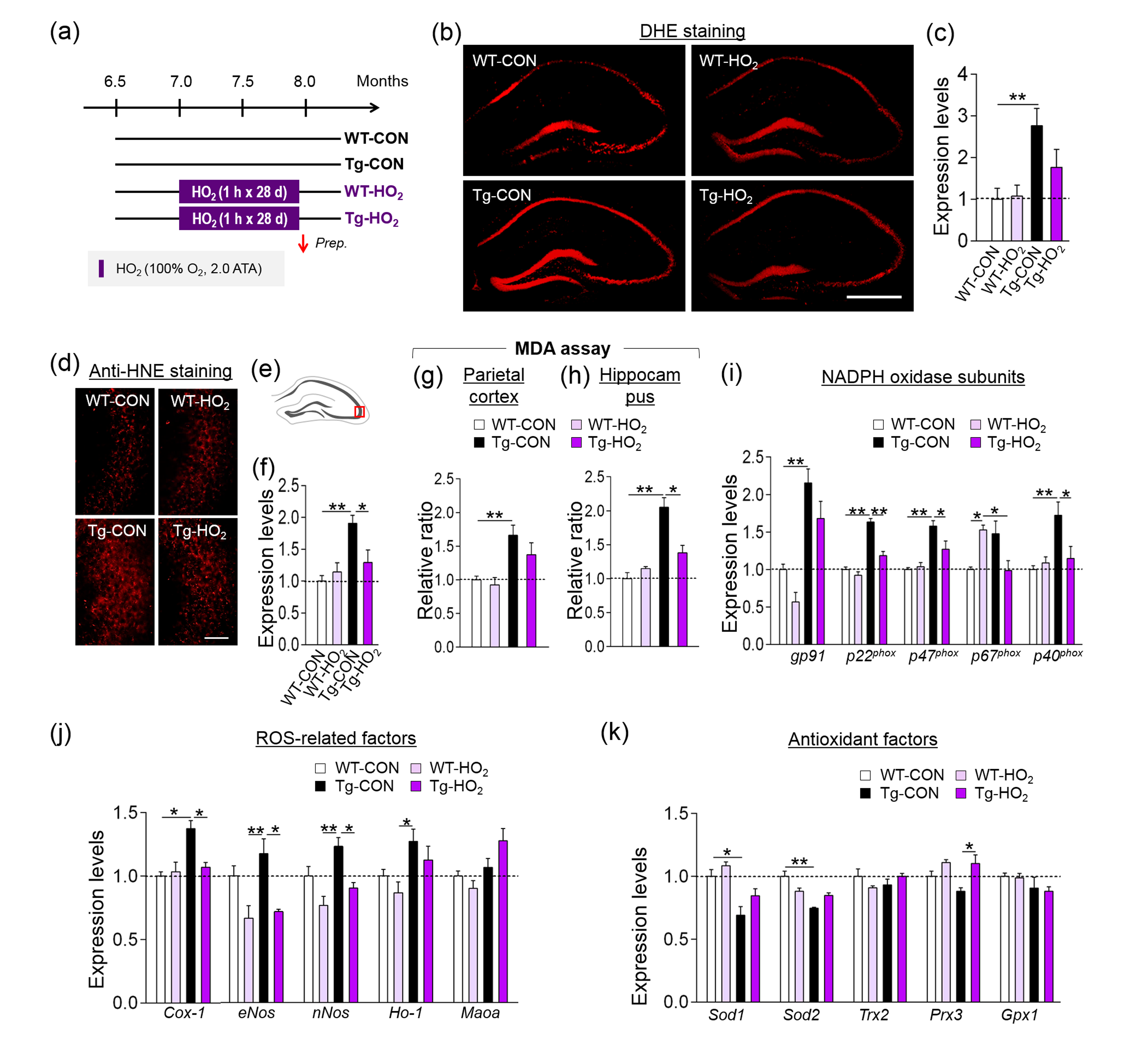

Supplement: Supplementary file 3 [file ACEL-18-e12888-s003.tif]

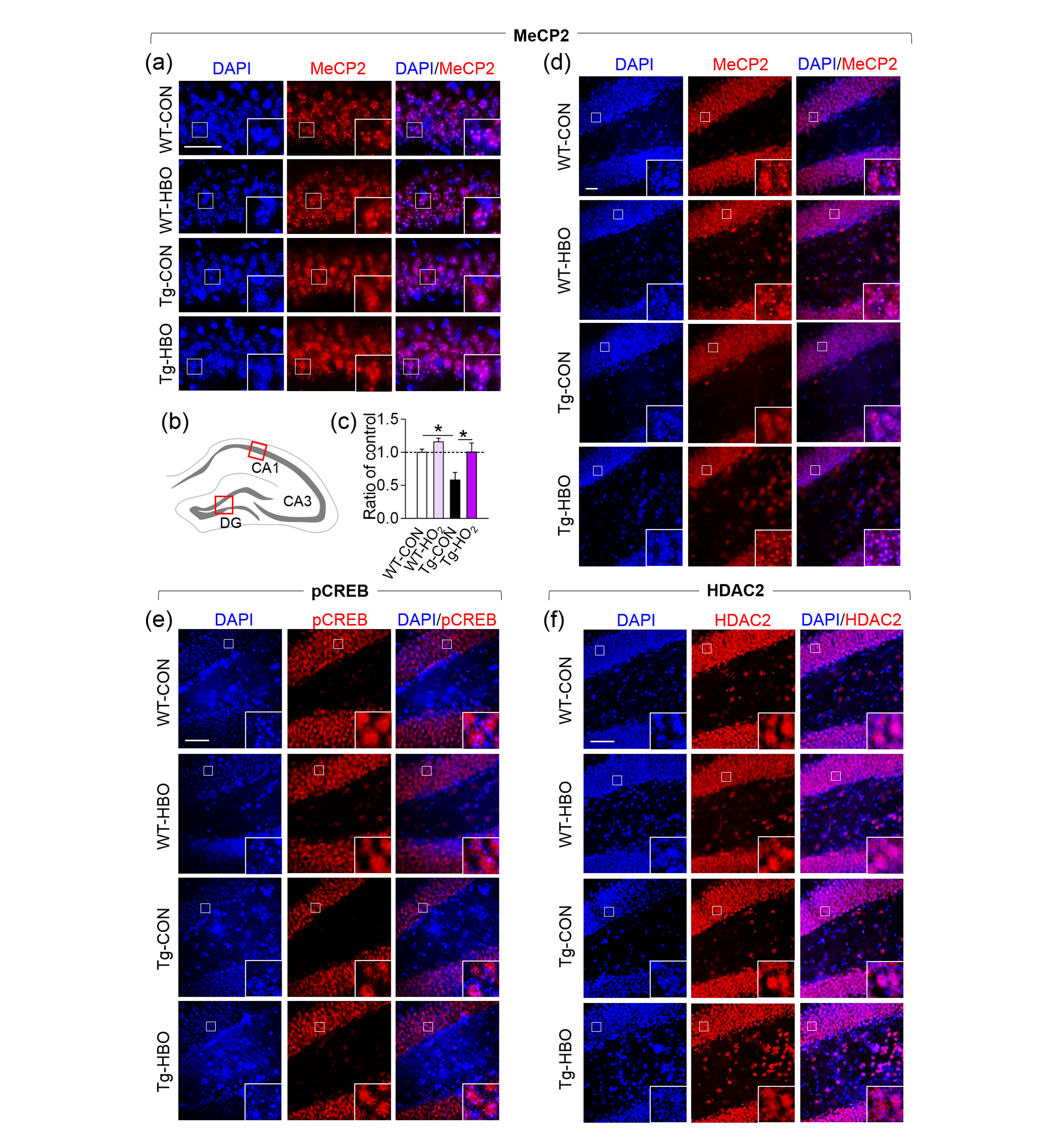

Supplement: Supplementary file 4 [file ACEL-18-e12888-s004.tif]

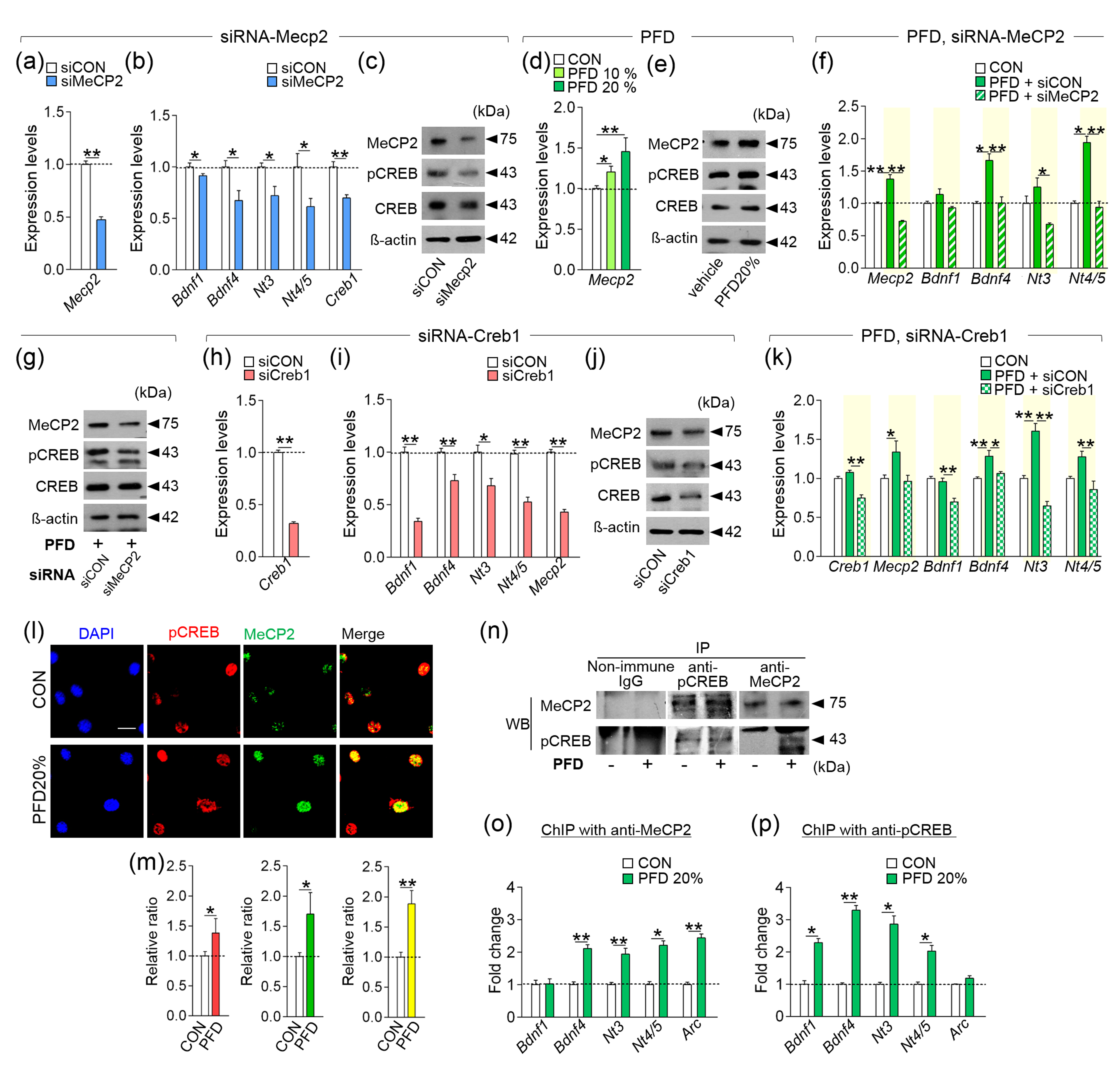

Supplement: Supplementary file 5 [file ACEL-18-e12888-s005.tif]

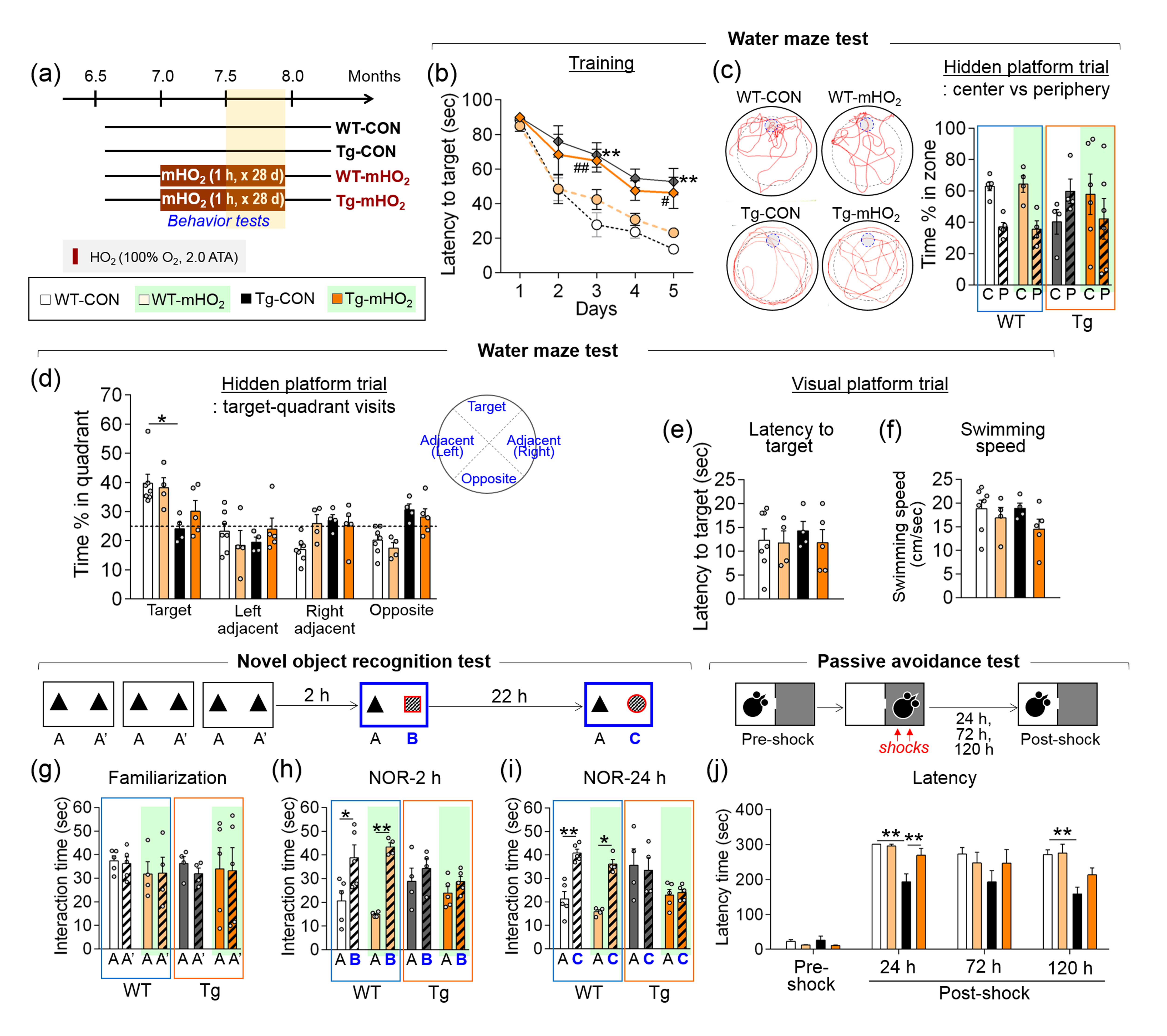

Supplement: Supplementary file 6 [file ACEL-18-e12888-s006.tif]
