# Supplementary material for: Hyperoxygenation revitalizes Alzheimer’s disease pathology through the upregulation of neurotrophic factors
Source: Aging Cell. 2019 Feb 11;18(2):e12888. doi: 10.1111/acel.12888 (PMC6413661; doi:10.1111/acel.12888)
Supplement: Supplementary file 8 [file ACEL-18-e12888-s008.docx]

**Hyperoxygenation revitalizes AD pathology through increase of neurotrophic factors**

Juli Choi^1^, Hye-Jin Kwon^1^, Jung-Eun Lee^1^, Yunjin Lee^1^, Ju-Young Seoh^2^ and

Pyung-Lim Han^1,3,*^

^1,3^Departments of Brain and Cognitive Sciences and ^3^Chemistry and Nano Science, Ewha Womans University, Seoul, Republic of Korea, 03760; ^2^Department of Microbiology, College of Medicine, Ewha Womans University, Seoul, Republic of Korea, 07985.

***Corresponding author**

**Pyung-Lim Han, Ph.D.**

Department of Brain and Cognitive Sciences, Ewha Womans University, 11-1 Daehyun-Dong, Seodaemoon-Gu, Seoul, 120-750, Republic of Korea.

Tel: +82-2-3277-4130, FAX: +82-2-3277-3419, E-mail : [plhan@ewha.ac.kr](mailto:plhan@ewha.ac.kr)

**Supplementary information**

Figures S1-6 with legends

Expended experimental procedures

**
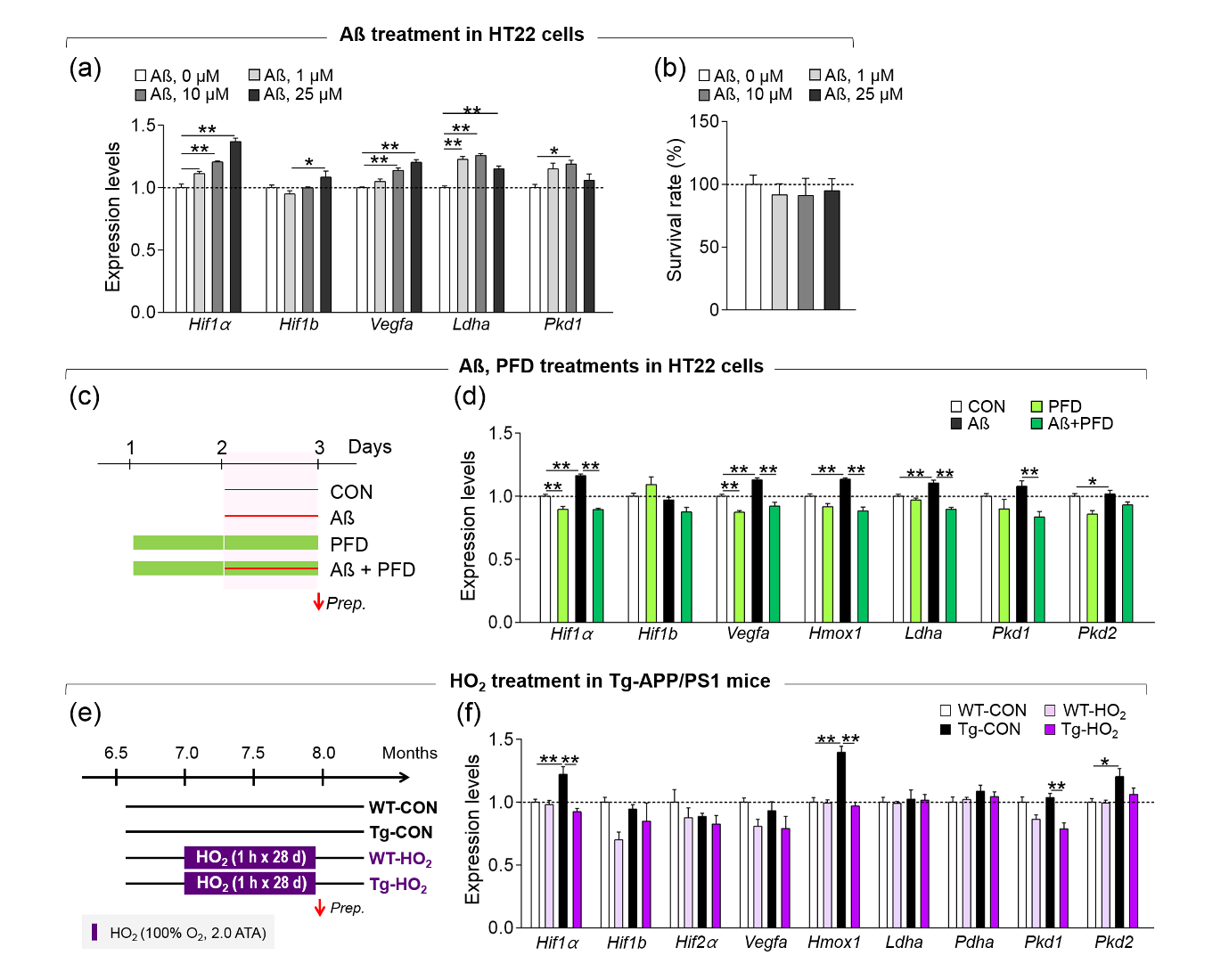
**

**Figure S1.** **Hyperoxygenation suppressed the expression of hypoxia-related genes in HT22 cells and in the hippocampus of Tg-APP/PS1 mice.** (**a**) Real-time PCR data showing the levels of *Hif-1α*, *Hif-1b*, *Vegfa*, *Ldha*, and *Pdk1* transcripts in HT22 cells treated with Aß42 (1, 10, and 25 μM) for 24 h. (**b**) The Aß42 doses (1, 10, and 25 μM) did not change the cell viability of HT22 cells, as assessed by a WST-1 assay. (**c** and **d**) Experimental design for the treatment of HT22 cells with Aß42 and perfluorodecalin (PFD) (c). Aß42 (25 μM) was treated for 24 h. PFD (green shadow) was given at 20%, starting 24 h prior to Aß42 treatment (red line). Arrow, time point for tissue prep. The PFD dose was chosen from a dose study. Real-time PCR data (d) showing the levels of *Hif-1α*, *Hif-1b*, *Vegfa*, *Hmox1*, *Ldha*, *Pdha*, *Pdk1*, and *Pdk2* transcripts in HT22 cells treated with Aß42, PFD, or Aß42 plus PFD for 24 h. (**e** and **f**) Experimental design (e). Tg-APP/PS1 and their wildtype control mice were treated with hyperoxygenation (HO_2_; 100% O_2_, 2 ATA) from 7 months of age for 1 h daily for 28 days. Arrow, time point for tissue prep. Real-time PCR data (f) showing the levels of *Hif-1α*, *Hif-1b*, *Vegfa*, *Hmox1*, *Ldha*, *Pdha*, *Pdk1*, and *Pdk2* transcripts in the hippocampus of wildtype mice (WT-CON), Tg-APP/PS1 mice (Tg-CON), wildtype mice treated with HO_2_ (WT-HO_2_), and Tg-APP/PS1 mice treated with HO_2_ (Tg-HO_2_). Data are presented as mean ± SEM. **p* < .05; ***p* < .01 (One-way ANOVA followed by Newman-Keuls *post hoc* test, and two-way ANOVA followed by Bonferroni *post hoc* test).

**
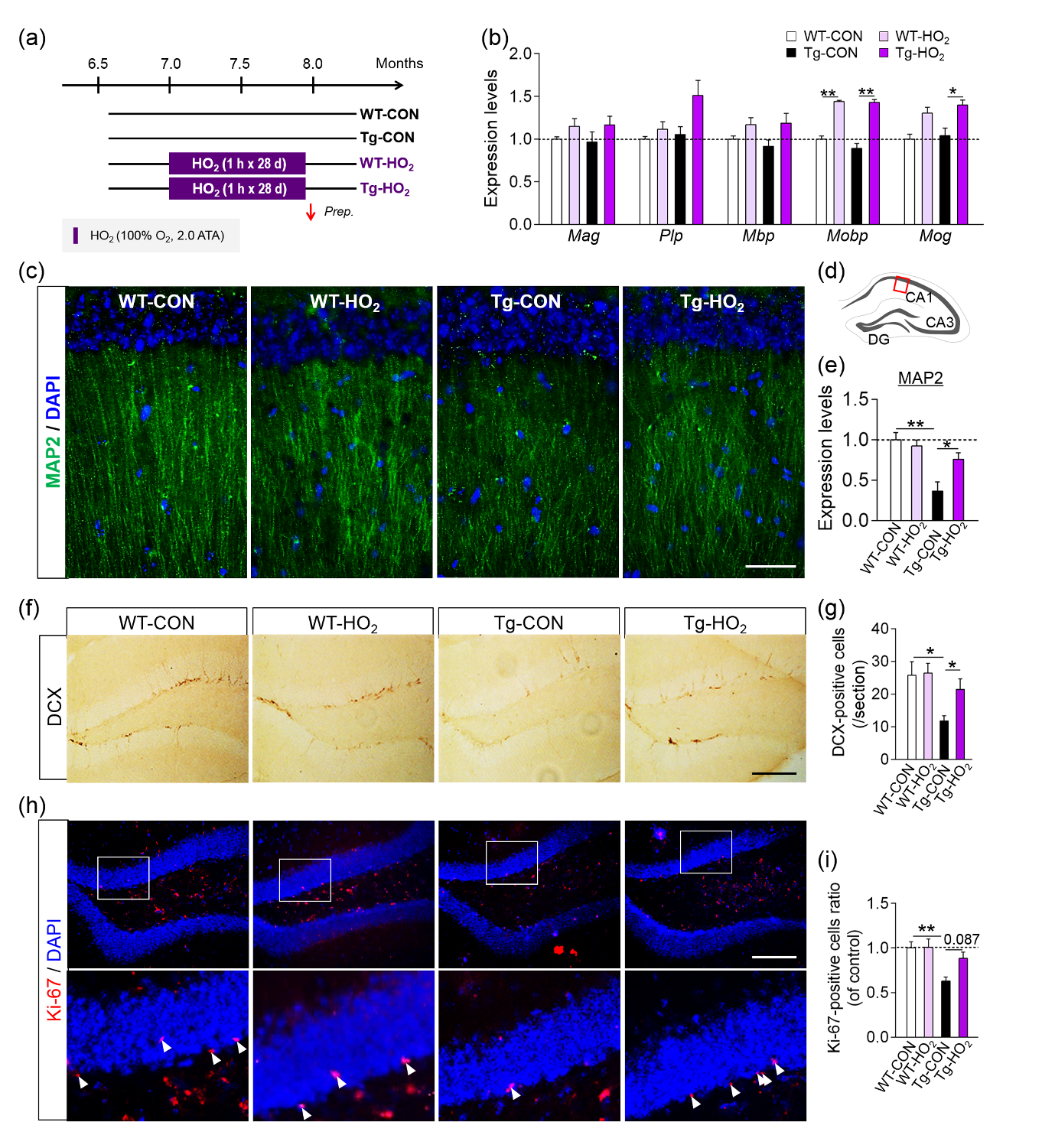
**

**Figure S2.** **HO_2_ treatment revived the neuritic atrophy and reduced neurogenesis in the hippocampus of Tg-APP/PS1 mice.** (**a**) Experimental design. Tg-APP/PS1 and their wildtype control were treated with hyperoxygenation (HO_2_; 100% O_2_, 2 ATA) for 1 h daily for 28 days. Arrow, time point for tissue prep. (**b**) Real-time PCR data showing expression levels of myelin**-**associated glycoprotein (*Mag*), proteolipid protein (*Plp*), myelin basic protein (*Mbp*), myelin**-**associated oligodendrocyte basic protein (*Mobp*), and myelin oligodendrocyte glycoprotein (*Mog*) in the hippocampus of WT-CON, WT- HO_2_, Tg-CON, and Tg-HO_2_. (**c-e**) Photomicrographs (c) showing anti-MAP2 staining in the CA1 subregion (d, marked with a box) of WT-CON, WT- HO_2_, Tg-CON, and Tg-HO_2_ mice. Quantification levels (e). MAP2, green; DAPI, blue. Scale bars, 50 μm. (**f-i**) Photomicrographs showing the dentate gyrus (DG) stained with anti-DCX antibody (f), anti-Ki-67 antibody (red), and DAPI (blue) (h) of WT-CON, WT- HO_2_, Tg-CON, and Tg-HO_2_ mice. High magnification of the boxed area in the DG stained with anti-Ki-67 antibody (h, bottom panels). Arrow heads, anti-Ki-67-positive cells. Quantification of DCX- (g) and Ki-67-stained cells (i). Scale bars, 100 μm. Data are presented as mean ± SEM. **p* < .05; ***p* < .01 (Two-way ANOVA followed by Bonferroni *post hoc* test).

**
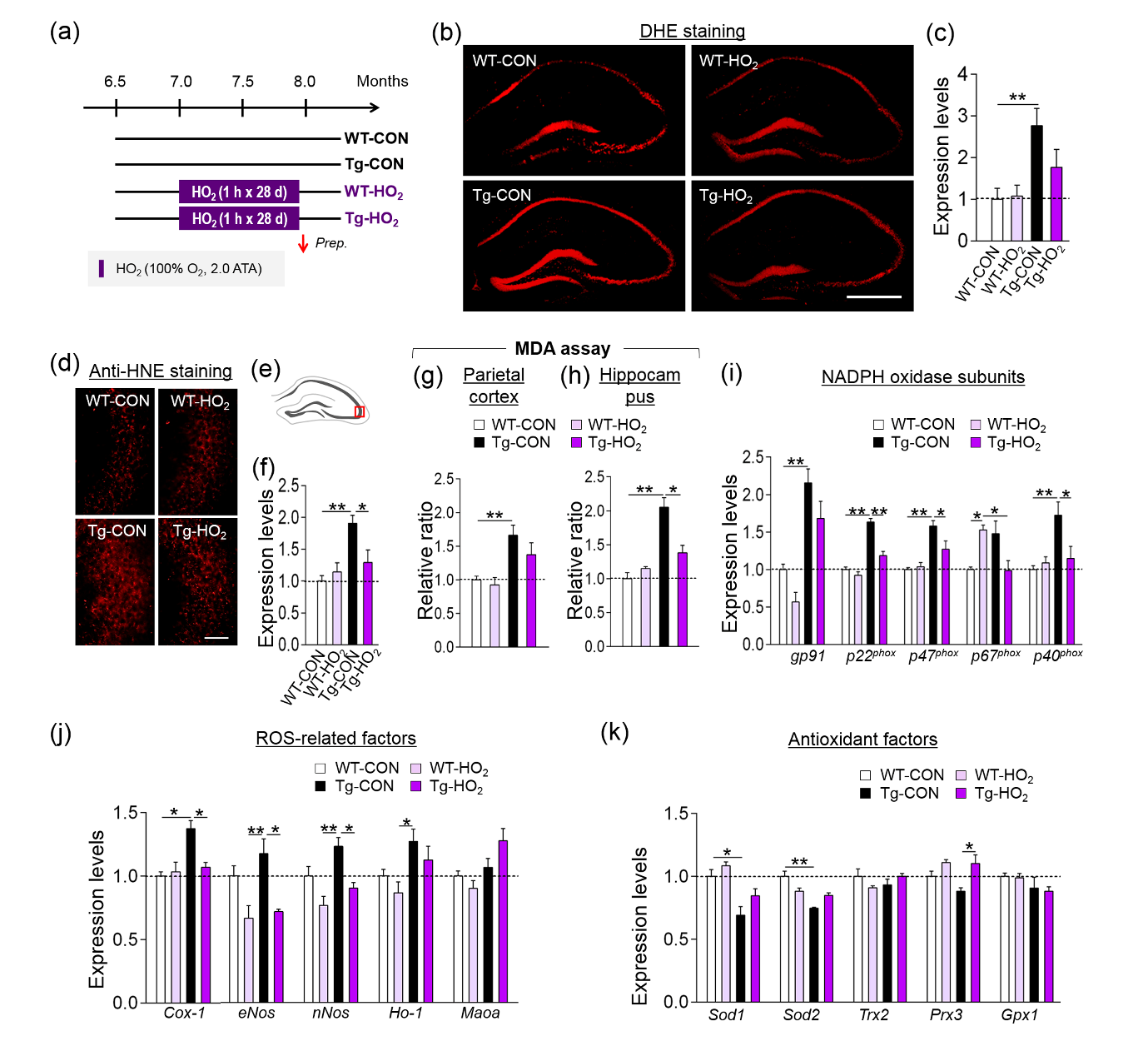
**

**Figure S3.** **HO_2_ treatment suppressed the ROS levels accumulated in the brain of Tg-APP/PS1 mice.** (**a**) Experimental design for HO_2_ treatment and ROS assessment. Mice were treated with HO_2_ (100% O_2_, 2 ATA) for 1 h daily for 28 days. WT-CON, wildtype mice; Tg-CON, Tg-APP/PS1 control mice; WT-HO_2_, wildtype mice treated with HO_2_; Tg-HO2, Tg-APP/PS1 mice treated with HO_2_. Arrow, time point for tissue preparation. (**b** and **c**) Representative photomicrographs showing dihydroethidium (DHE)-stained ROS levels in the hippocampus (b) of WT-CON, WT-HO_2_, Tg-CON, and Tg-HO_2_. Quantification levels (c). Scale bar, 1 mm. (**d-f**) Representative photomicrographs showing anti-HNE (4-hydroxynonenal)-stained pyramidal neurons (d) in the CA3 region (marked with a box; e) of WT-CON, WT-HO_2_, Tg-CON, and Tg-HO_2_. Quantification levels (f). Scale bar, 50 μm. (**g** and **h**) Malondialdehyde (MDA) levels in the parietal cortex (g) and hippocampus (h) of WT-CON, WT-HO_2_, Tg-CON, and Tg-HO_2_. (**i-k**) Real-time PCR data showing expression levels of the NADPH oxidase subunits (*gp91*, *p22^phox^*, *p47^phox^*, *p67^phox^*, and *p40^phox^*) (i), *Cox-1*, *eNoa*, *nNos*, *Ho-1*, *Maoa* (j), *Sod1*, *Sod2*, *Trx2*, *Prx3*, and *Gpx1* (k) in the hippocampus of WT-CON, WT-HO_2_, Tg-CON, and Tg-HO_2_. Data are presented as mean ± SEM. **p* < .05; ***p* < .01 (Two-way ANOVA followed by Bonferroni *post hoc* test).

**
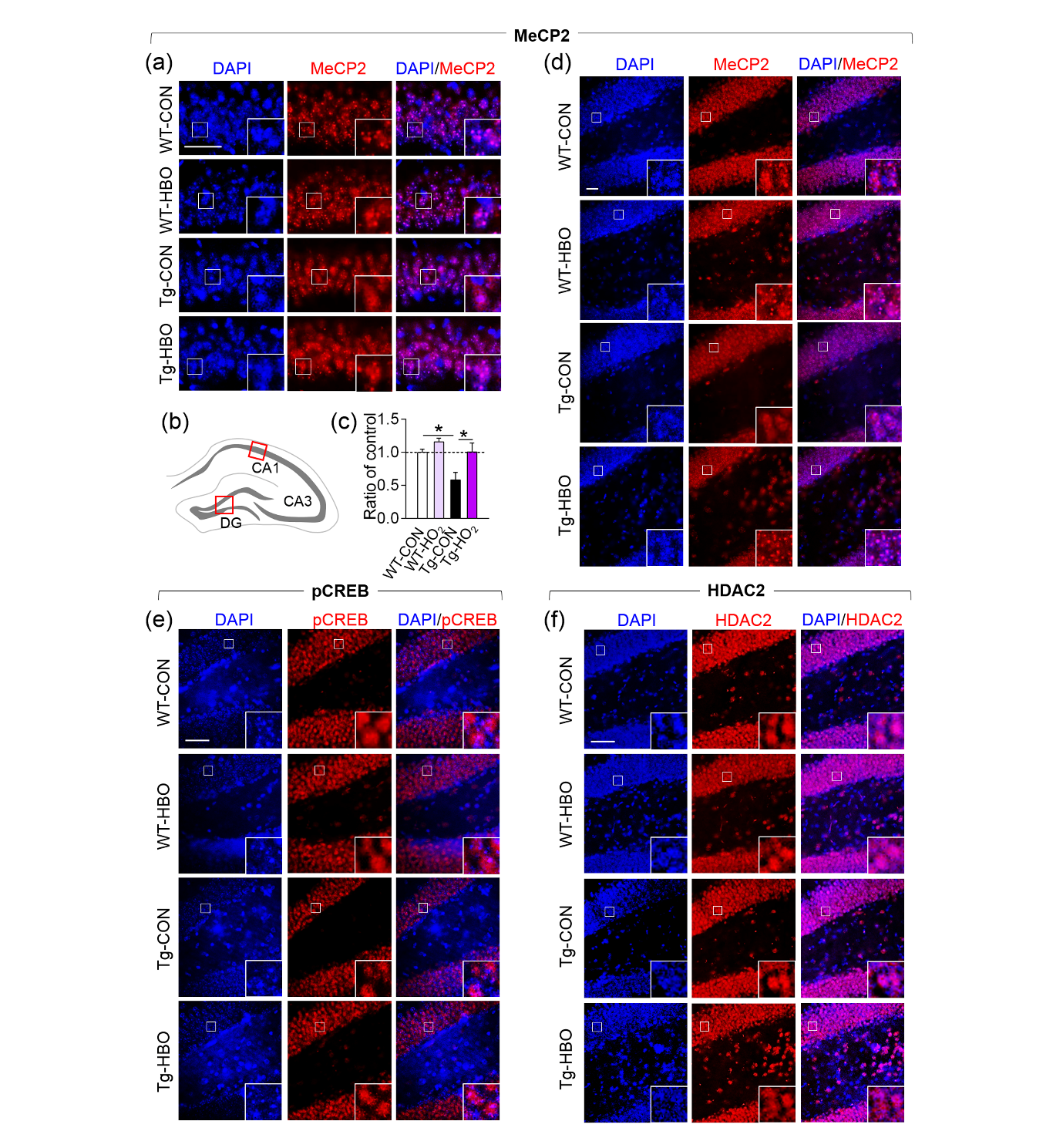
**

**Figure S4.** **Effects of HO_2_ on the expression of MeCP2, pCREB, and HDAC2 in the hippocampus of Tg-APP/PS1 mice.** (**a-d**) Photomicrographs showing MeCP2 expression in the CA1 (a) and dentate gyrus (d) regions of the hippocampus (b) of WT-CON, WT-HO_2_, Tg-CON, and Tg-HO_2_. Insets in (b), high magnification of the area marked with a box in the dentate gyrus (DG). Anti-MeCP2 antibody, red; DAPI, blue. Quantification of MeCP2 expression levels in the CA1 (c). Scale bars, 50 μm. (**e**) Photomicrographs showing pCREB expression in the dentate gyrus (DG) of WT-CON, WT-HO_2_, Tg-CON, and Tg-HO_2_. Insets, high magnification of the area marked with a box. Anti-pCREB antibody, red; DAPI, blue. Scale bar, 100 μm. (**f**) Photomicrographs showing HDAC2 expression in the dentate gyrus (DG) of WT-CON, WT-HO_2_, Tg-CON, and Tg-HO_2_. Insets, high magnification of the area marked with a box. Anti-HDAC2 antibody, red; DAPI, blue. Scale bar, 100 μm. Data are presented as mean ± SEM. **p* < .05 (Two-way ANOVA followed by Bonferroni *post hoc* test).

**
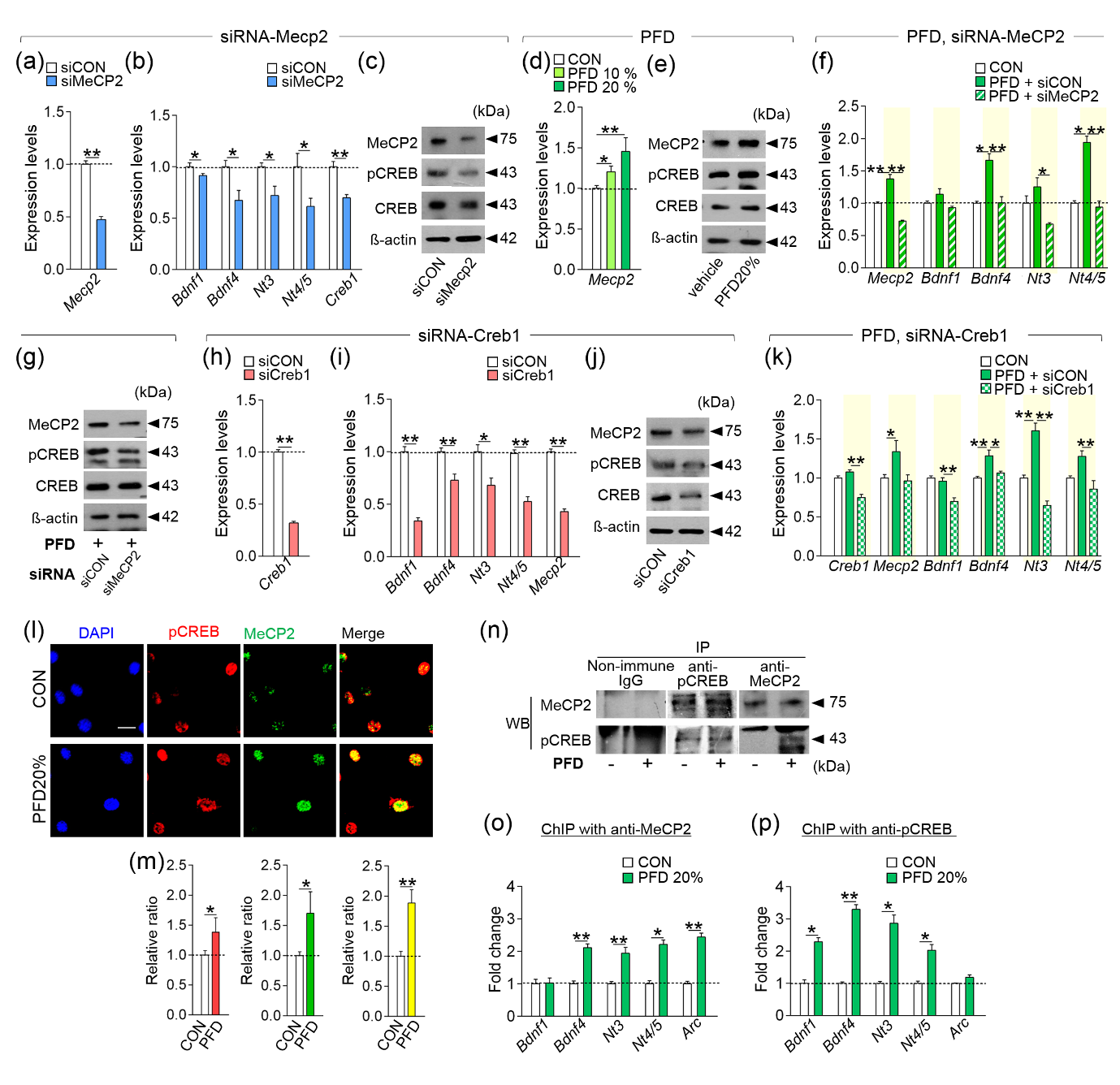
**

**Figure S5.** **The induction of neurotrophic factors by hyperoxygenation in HT22 cells was mediated by MeCP2/pCREB activity.** (**a**-**c**) Real-time PCR data showing expression levels of *Mecp2* (a), *Bdnf1*, *Bdnf4*, *Nt3*, *Nt4/5*, and *Creb1* (b) and Western blots showing expression levels of pCREB and MeCP2 (c) in HT22 cells transfected with siRNA-MeCP2 or siRNA-control. (**d** and **e**) Real-time PCR data showing expression levels of *Mecp2* (d) in HT22 cells treated with PFD (10, 20%) and Western blots showing expression levels of pCREB and MeCP2 (e) in HT22 cells treated with PFD (20%). (**f** and **g**) Real-time PCR data showing expression levels of *Mecp2*, *Bdnf1*, *Bdnf4*, *Nt3*, and *Nt4/5* (f) and Western blots showing expression levels of MeCP2 and pCREB (g) in HT22 cells treated with PFD (20%) and transfected with siRNA-MeCP2. PFD (20%)-induced increase in the expression of *Bdnf1*, *Bdnf4*, *Nt3*, *Nt4/5* was suppressed by siRNA-mediated inhibition of *Mecp2* (f). (**h**-**j**) Real-time PCR data showing expression levels of *Creb1* (h), *Bdnf1*, *Bdnf4*, *Nt3*, *Nt4/5*, and *Mecp2* (i) and Western blots showing expression levels of pCREB and MeCP2 (j) in HT22 cells transfected with siRNA-Creb1 or siRNA-control. (**k**) Real-time PCR data showing expression levels of *Creb1*, *Mecp2*, *Bdnf1*, *Bdnf4*, *Nt3*, and *Nt4/5* in HT22 cells treated with PFD (20%) and transfected with siRNA-Creb1. (**l** and **m**) Immunofluorescent images showing HT22 cells stained with anti-pCREB (red), anti-MeCP2 (green), and DAPI (blue). PFD (20%) treatment increased the levels of MeCP2 and pCREB, and their nuclear co-localization (l). Quantification of staining levels of pCREB, MeCP2, and merged images (m). Scale bars, 20 μm. (**n**) Western blots showing co-immunoprecipitation of MeCP2 and pCREB in HT22 cells in the presence or absence of PFD (10%). (**o** and **p**) ChIP assay showing the levels of MeCP2 (o) and pCREB (p) binding to the promoter of *Bdnf1*, *Bdnf4*, *Nt3*, *Nt4/5* in HT22 cells treated with PFD (20%). The Arc promoter was used as a positive control for MeCP2 binding. The maps in Fig. 3A depicting the promoter structure and the regions of *Bdnf1*, *Bdnf4*, *Nt3*, *Nt4/5* used for PCR analysis were applicable here. Data are presented as mean ± SEM. *p < .05; **p < .01 (Student’s *t*-test, and one-way ANOVA followed by Newman-Keuls *post hoc* test).

**
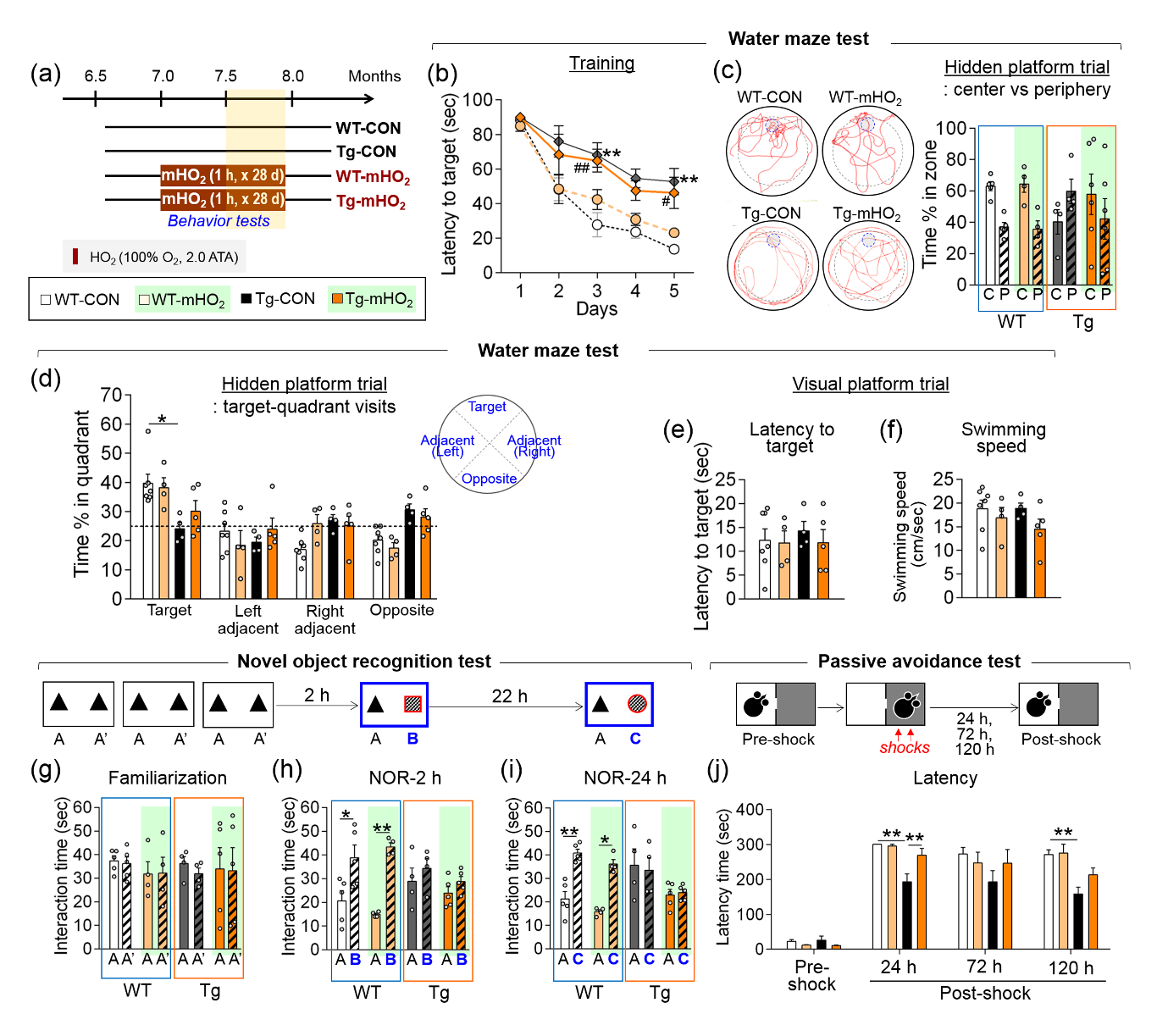
**

**Figure S6.** **Treatment with moderate hyperoxygenation was not sufficient to improve the cognitive deficits of Tg-APP/PS1 mice.** (**a**) Experimental design for treatment of mice with moderate hyperoxygenation (mHO_2_). Mice were treated with mHO_2_ (42% O_2_, 2 ATA) from 7 months of age for 1 h daily for 28 days, in a way in a manner similar to that used for HO_2_ (100% O_2_, 2 ATA) in Figure 5. The behavioral tests were performed in the following order: water maze test, novel object recognition test, and passive avoidance test. The color codes in the figure legends for WT-CON, WT-mHO_2_, Tg-CON, and Tg-mHO_2_ mice are applicable to all figures. (**b-d**) The effects of mHO_2_ on spatial memory and associated behaviors in the water maze test. The latency to finding the hidden platform (b) during the training period for WT-CON, WT-mHO_2_, Tg-CON, and Tg-mHO_2_. The percent time spent in the periphery *vs*. center (c) and in each quadrant (d) of the water maze in the hidden platform trial. Representative tracking of each group during the probe trial (c). Dashed line on (d) indicates a 25% chance of finding the platform. C, center; P, periphery. Diagram with quadrants: T, target; L, left; R, right; O, opposite. (**e** and **f**) The latency to finding the visual platform (e) and swim speed (f) during the visual platform trial of the water maze test for WT-CON, WT-mHO_2_, Tg-CON, and Tg-mHO_2_. (**g-i**) The effects of mHO_2_ on novel object recognition memory. Diagrams depicting the experimental design and the NOR test steps. Time spent exploring the two identical objects during the familiarization (10-min exposure, three repeats) (g) and between a new and an old object 2 h after familiarization (h, NOR-2h) and 24 h after familiarization (i, NOR-24h). The exploration time during the first session of the familiarization phase is presented. A, B and C represent different objects. (**j** and **k**) The effects of mHO_2_ on fear memory in the passive avoidance test. Diagrams depicting the experimental design for the PA test (j). The latency to entering the dark chamber at the pre-shock, and 24 h and 72 h after shock (k). Data are presented as mean ± SEM. WT-CON, n = 5-8; WT-mHO_2_, n = 4; Tg-CON, n = 4-5; Tg-mHO_2_, n = 5-6 per group. **p* < .05; ***p* < .01, difference between indicated groups; #*p* < .05; ##*p* < .01, difference between Tg-CON and Tg-mHO_2_ (Two-way ANOVA followed by Bonferroni *post hoc* test, and two-way Repeated Measures ANOVA followed by Bonferroni *post hoc* test).

**Expended experimental procedures**

**Animals**

Tg-APPswe/PS1dE9 (Tg-APP/PS1) mice show plaque deposition from 6.5 months of age and severe cognitive deficits at 7-7.5 months of age (Jankowsky et al, 2001; Savonenko et al., 2005; Kim et al., 2012). Tg-APP/PS1 mice were crossed with C57BL6 × C3H hybrid mice as described previously (Seo et al. 2011) or independently with C57BL/6 mice for more than 10 generations. From weaning, female and male mice were housed separately, with 2-3 animals per cage under a 12 h light/dark cycle (lights on at 7 a.m.) in a controlled room at 50–60% humidity and a temperature of 23°C.

Genotyping of Tg-APP/PS1 mice was performed by genomic PCR using the following primers: 5’- CTAGGCCACAGAATTGAAAGATCT-3’ and 5’- GTAGGTGGAAATTCTAGCATCATCC-3’ for wildtype (324 bp), 5’-AATAGAGAACGGCAGGAGCA-3’ and 5’- GCCATGAGGGCACTAATCAT-3’ for the PS1 gene (324 / 608 bp), 5’-AGGACTGACCACTCGACCAG-3’ and 5’- CGGGGGTCTAGTTCTGCAT-3’ for the APP gene (324 /350 bp). All animals were handled in accordance with the animal care guidelines of the Ewha Womans University, and all animal experiments were approved by the Ewha Womans University Institutional Animal Care and Use Committee (IACUC 16-019).

**HT22 cell culture and drug treatment**

HT22 mouse hippocampal cells were cultured as described previously (Seo et al., 2012). Briefly, HT22 cells were maintained in Dulbecco’s modified Eagle’s medium (DMEM; LM001-05, Welgene, Gyeongan-si, Korea) supplemented with 10% heat-inactivated fetal bovine serum (FBS; FB02-500, Serum Source International, Charlotte, NC, USA) and penicillin (20 units/ml)/streptomycin (20 mg/ml) (LS020-02, Welgene) at 37°C and 5% CO_2_. When HT22 cells reached 70-80% confluency, they were trypsinized, stained with 0.4% trypan blue, and counted using a hemocytometer. They were then plated at 1.0 × 10^5^ cells/well in a 6-well plate or 1.0 × 10^6^ cells in 100-mm dish, grown in DMEM media containing 10% FBS for 24 h, and then subjected to drug treatment as described below.

Aß(1-42) was prepared as described previously (Seo et al. 2011). Briefly, Aß(1-42) (03112, Invitrogen, Camarillo, CA, USA) was dissolved in 1X PBS (137 mM NaCl, 2.7 mM KCl, 10 mM Na_2_HPO_4_, 1.8 mM KH_2_PO_4_) to the concentration of 100 μM and incubated at 37°C for 1 week with gentle rocking. Aggregated Aß(1-42) was stored at -80°C and diluted with 1X PBS into the concentration as needed (e.g., 1, 10, and 25 μM). HT22 cells were treated with Aß(1-42) and/or perflurodecalin (PFD) (P9900, Sigma-Aldrich, St. Louis, MO, USA) at indicated concentrations in DMEM media containing 1% FBS without penicillin/streptomycin for 24 h and harvested for analyses.

**Cell survival assay**

Cell survival assessment was carried out using an EZ-Cytox cell viability assay kit (EZ1000, Daeil Lab Service Co., LTD, Guro-gu, Seoul, Korea) in a manner that was based on the WST-1 (Water soluble tetrazolium salt-1) assay method. HT22 cells reached 70-80% confluency were trypsinized, stained with trypan blue and plated at the density of 1.0 × 10^4^ cells/ml in 96-well plates (SPL Life Science, Pocheon-si, Korea). HT22 cells were treated with maturated Aß(1-42) at 1, 10, and 25 μM for 24 h in DMEM media containing 1% FBS without antibiotics. Cultured media (100 μl/well) was dispensed into a new 96-well plate, an assay agent from the EZ-Cytox kit was added to each well (10 μl/well), and plates were then incubated in a 37°C incubator for 30 min. The reaction product was assessed by measuring the absorbance at 450 nm using a VersaMax ELISA microplate reader (Molecular Devices, San Jose, CA, USA).

**Hyperoxygenation treatment in mice**

Mice were treated with hyperoxygenation (HO_2_) using a hyperbaric chamber (Particla Ltd., Daejeon, South Korea) as described previously (Kim et al., 2014) with a minor modification. Mice housed with 2-3 animals in a clear plastic mini-cage (18 x 12 x 14 cm) were placed in the hyperbaric chamber and exposed to 100% oxygen daily for 60 min at 2.0 ATA for 28 days or the indicated number of days (Figures 1A; Figures S1H). The desired pressure in the chamber was slowly reached by supplying the chamber with 100% O_2_ at 5 L/min at 2.0 ATA for 12 min, followed by continual replenishment with 100% O_2_ at 1 L/min for 60 min. Decompression was achieved at a rate of 1.5 L/min for over 40 min. During the HO_2_ treatment, oxygen level and temperature changes inside the chamber were monitored. HO_2_ treatment was initiated at 10:00 a.m. On the day of the behavioral test, HO_2_ treatment was given after the behavioral test was administered. For moderate hyperoxygenation (mHO_2_), mice were treated as above, but with atmospheric air, instead of 100% oxygen.

**Thioflavin S staining of Aß deposition in the brain**

Tg-APP/PS1 mice were individually anesthetized with 2.5% avertin (intraperitoneal injection, 20 μg/g body weight) and perfused with 0.9% saline via a trans-cardiac method. The left hemisphere of each mouse was post-fixed with 4% paraformaldehyde in 0.1 M phosphate buffer, pH 7.4, at 4°C overnight, while the right hemisphere was used for an Aß ELISA assay as described below. Each fixed hemisphere was coronally cut into 40-μm-thick sections with a vibratome (Leica VT 1000S, Leica Instruments, Mussloch, Germany).

Free-floating brain sections were washed with 1X PBS (137 mM NaCl, 2.7 mM KCl, 10 mM Na_2_HPO_4_, 1.8 mM KH_2_PO_4_) three times for 10 min and mounted onto a glass slide. Thioflavin S (ThS; T1892, Sigma-Aldrich) was dissolved in 50% ethanol and additionally in H_2_O to a concentration of 1 mM. Brain sections on the glass slide were incubated with 1 mM ThS for 5 min. To quench nonspecific binding, stained sections were washed in 100, 95, and 50% ethanol for 30 s each and then rinsed with 1X PBS twice. The sections were lightly dried and coverslipped with anti-fade fluorescent mounting medium (S3023, DAKO, Carpinteria, CA, USA).

The images of plaques were photographed using an Olympus BX51 microscope equipped with a DP71 camera equipped with a fluorescent filter set. The number of plaques and size of the stained area of plaques were analyzed using the MetaMorph Microscopy Automation & Image Analysis software (Molecular Devices, Sunnyvale, CA, USA).

**ELISA assay of Aß accumulation**

ELISA assessments of Aß(1-40) and Aß(1-42) levels were carried out as described previously (Seo et al., 2009; Kim et al., 2012). Briefly, Tg-APP/PS1 mice were sacrificed and perfused with 0.9% saline. The right hemisphere of each mouse brain was used for ELISA assays, while the left hemisphere was used for thioflavin S staining, as stated above. The prefrontal cortex and hippocampus of the right hemisphere were separately homogenized in Tris-buffered saline (20 mM Tris-Cl, 137 mM NaCl, pH 7.6) containing a proteinase inhibitor cocktail (11836153001, Roche, Indianapolis, IN, USA), followed by sonication on ice with 15-sec pulses and 30-sec intervals, repeated three times using an Epishear probe sonicator at a 40% power outlet (Active Motif, Carlsbad, CA, USA).

The homogenates were centrifuged at 100,000 g at 4°C for 1 h and the supernatant was used to assess Tris buffer-soluble forms of Aß. The pellet was resuspended in ice-cold 70% formic acid (FA) and sonicated under the conditions described above. After incubating on ice for 30 min, the homogenates were centrifuged at 100,000 g at 4°C for 1 h. The supernatant was collected and neutralized with 1 M Tris–HCl buffer (pH 11) at a 1:20 dilution, and the diluted formic acid extract was used for the assessment of formic acid-extractable Aß. Measurement of Aß(40) and Aß(42) levels was performed using Aß40 or Aß42 ELISA kits (KHB3482 for Aß40 and KHB3442 for Aß42, Invitrogen) by following the manufacturer’s instructions.

**Quantitative real-time PCR**

Quantitative real-time PCR analysis was performed as described previously (Choi et al., 2015; Kim et al., 2015). The prefrontal cortex and hippocampus were separately decomposed using pellet pestles (Z359971, Sigma-Aldrich). Total RNA was isolated from tissue homogenates or HT22 cells using TRIzol reagent (15596-018, Invitrogen). After treating with DNase I to avoid genomic DNA contamination, 2 μg of total RNA was converted to cDNA using a reverse transcriptase system (Promega, Madison, WI, USA).

Real-time PCR was performed with 4 μl of cDNA (1/8 dilution of the converted cDNA), 10 μl of 2X iQTM SYBR Green Supermix (Bio-Rad Laboratories, Foster City, CA, USA), and 1 μl each of 5 pmol/μl forward and reverse primers in a volume of 20 μl using the CFX 96 Real-Time PCR System Detector (Bio-Rad Laboratories).

The primers used were: *Bdnf* (total form), forward 5’-TGGCTGACACTTTTGAGCAC-3’ and reverse 5’-GTTTGCGGCATCCAGGTAAT-3’; *Bdnf1 (for in vivo sample)*, forward 5’-CCTGCATCTGTTGGGGAGAC-3’ and reverse 5’-GCCTTGTCCGTGGACGTTTA-3’; *Bdnf1 (for in vitro sample)*, forward 5’- CAGTGACAGGCGTTGAGAAA-3’ and reverse 5’- AACGCCCTCATTCTGAGAGA-3’; *Bdnf4*, forward 5’-CAGAGCAGCTGCCTTGATGTT-3’ and reverse 5’-GCCTTGTCCGTGGACGTTTA-3’; *Cbp*, forward 5’-GGTTGCCTATGCTAAGAAAGT-3’ and reverse 5’-GATGCCTTGCTTATGTAAACG-3’; *Cox-1*, forward 5’-ACAGTATCACCTGCGGCTCT-3’ and reverse 5’-GGAAGCAACCCAAACACCT-3’; *Creb1*, forward 5’-TGGACAGCAGATTCTAGTG-3’ and reverse 5’-GGAGGACGCCATAACAAC-3’; *gp91^phox^*, forward 5’-ACAACTGGACAGGAACCTCA-3’ and reverse 5’-TCACCGATGTCAGAGAGAGC-3’; *Gpx1*, forward 5’-TTTCCCGTGCAATCAGTTC-3’ and reverse 5’-TCGGACGTACTTGAGGGAAT-3’; *Hdac1*, forward 5’-CAGTGTGGCTCAGATTCCCT-3’ and reverse 5’-GGGCAGCTCATTAGGGATCT-3’; *Hdac2*, forward 5’- GGGACAGGCTTGGTTGTTTC-3’ and reverse 5’-GAGCATCAGCAATGGCAAGT-3’; *Hdac3*, forward 5’-AGAGAGGTCCCGAGGAGAAC-3’ and reverse 5’-ACTCTTGGGGACACAGCATC-3’; *Hdac4*, forward 5’-CAATCCCACAGTCTCCGTGT-3’ and reverse 5’-CAGCACCCCACTAAGGTTCA-3’; *Hdac5*, forward 5’-TGTCACCGCCAGATGTTTTG-3’ and reverse 5’-TGAGCAGAGCCGAGACACAG-3’; *Hif1b*, forward 5’-GCATGGGCTCACGAAGGT-3’ and reverse 5’-AACAGGGTCCACGGAGCTAGT-3’; *Hif1α*, forward 5’-ATCTCGGCGAAGCAAAGAGTC-3’ and reverse 5’-TGGGGAAGTGGCAACTGAT-3’; *Hif2α*, forward 5’-CTGAGGAAGGAGAAATCCCGT-3’ and reverse 5’-TGTGTCCGAAGGAAGCTGATG-3’; *Hmox1*, forward 5’-GGCGTCACTTCGTCAGAG-3’ and reverse 5’-ACTGGAGGAGCGGTGTC-3’; *Mag*, forward 5’-GGTGTTGAGGGAGGCAGTTG-3’ and reverse 5’-CGTTCTCTGCTAGGCAAGCA-3’; *Maoa*, forward 5’-TGTTGATGCACCATGGCAAG-3’ and reverse 5’-ACACTGCCTCACATACCACA-3’; *Mbp*, forward 5’-ACACACGAGAACTACCCATTATGG-3’ and reverse 5’-AGAAATGGACTACTGGGTTTTCATCT-3’; *Mecp2*, forward 5’-ACAGCGGCGCTCCATTATC-3’ and reverse 5’-CCCAGTTACCGTGAAGTCAAAA-3’; *Mobp*, forward 5’-AACTCCAAGCGTGAGATCGT-3’ and reverse 5’-CTCGGTCACTTCTTCCTTGG-3’; *Mog*, forward 5’-CTGTTTGTTATTGTGCCTGTTCTTG-3’ and reverse 5’-AGTCTTCGGTGCAGCCAGTT-3’, *eNos*, forward 5’-GAGAGCGAGCTGGTGTTTG-3’ and reverse 5’-TGATGGCTGAACGAAGATTG-3’; *nNos*, forward 5’-ACCAGCTCTTCCCTCTAGCC-3’ and reverse 5’-GCCATAGATGAGCTCCGTGT-3’; *Nt3*, forward 5’-TACTACGGCAACAGAGACG-3’ and reverse 5’-GTTGCCCACATAATCCTCC-3’; *Nt4/5*, forward 5’-AGCGTTGCCTAGGAATACAGC-3’ and reverse 5’-GGTCATGTTGGATGGGAGGTATC-3’; *p22^phox^*, forward 5’-CCCCGGGGAAAGAGGAAAAA-3’ and reverse 5’-AGGACAGCCCGGACGTAGTA-3’; *p300*, forward 5’-GACTGAGCAGCGATAATG-3’ and reverse 5’-CAAGGTGTCTCTAGTGTATG-3’; *p40^phox^*, forward 5’-TGGCCCAGCAGCTGCG-3’ and reverse 5’-CGTAGCGGCGGTAGATGAG-3’; *p47^phox^*, forward 5’-ACAGAGTCATCCCACACCTC-3’ and reverse 5’-GTGGGCAGTTTCAGGTCATC-3’; *p67^phox^*, forward 5’-GGGAACCAGCTGATAGACTA-3’ and reverse 5’-TCCATTCCTCYTTCTTGGCA-3’; *Pdha*, forward 5’-CTGGCATAAACCCTACGGAC-3’ and reverse 5’-CGCCTTTCCCTTTAGCAC-3’; *Pdk1*, forward 5’-ATACACTGCCAATGATTGAC-3’ and reverse 5’-GCCATGCCGCTGTAAC-3’; *Pdk2*, forward 5’-TACCTCAGCCGCATCTC-3’ and reverse 5’-TGGCGTTGGTGGCATTGAC-3’; *Plp*, forward 5’-CCCACCCCTATCCGCTAGTT-3’ and reverse 5’-CAGGAAAAAAAGCACCATTGTG-3’; *Prx3*, forward 5’-GAACCTGTTTGACAGACATACTGTG-3’ and reverse 5’-GGGGTGTGGAAAGAGGAACT-3’; *Rest*, forward 5’-TGAGGGAGAGTTTGTGTGTAT-3’ and reverse 5’-AGTGGCGATTGAGGTGTT-3’; *Sirt1*, forward 5’-GATCCTTCAGTGTCATGGTTC-3’ and reverse 5’-ATGGCAAGTGGCTCATCA-3’; *Sod1*, forward 5’-TGCGTGCTGAAGGGCGAC-3’ and reverse 5’-GTCCTGACAACACAACTGGTTC-3’; *Sod2*, forward 5’-GGAGCAAGGTCGCTTACAGA-3’ and reverse 5’-GTGCTCCCACACGTCAATC-3’; *Trka*, forward 5’-GTCATGGCTGCTTTTATGG-3’ and reverse 5’-ACTGGCGAGAAGGAGACAG-3’; *Trkb*, forward 5’-AAGGACTTTCATCGGGAAGCTG-3’ and reverse 5’-TCGCCCTCCACACAGACAC-3’; *Trx2*, forward 5’-ATCCCTCTGCTCGCACTG-3’ and reverse 5’-AAGCCCACACACCCTGAG-3’; *Vegfa*, forward 5’-GCAGGCTGCTGTAACGATGAA-3’ and reverse 5’-TTTGATCCGCATGATCTGCAT-3’; *Gapdh*, forward 5’-AGAAGGTGGTGAAGCAGGCATC-3’ and reverse 5’-CGAAGGTGGAAGA GTGGGAGTTG-3’; *L32*, forward 5'-GCTGCCATCTGTTTTACGG-3' and reverse 5'-TGACTGGTGCCTGATGAACT-3'.

**Western blot analysis**

Western blot analyses were carried out as described previously (Choi et al. 2015; Kim et al., 2015). Briefly, the prefrontal cortex and hippocampus were separately homogenized in homogenization buffer (50 mM Tris–HCl, pH 8.0, 150 mM NaCl, 1% Nonidet P-40, 0.1% SDS, and 0.1% sodium deoxycholate) containing a protease inhibitor cocktail (Roche) by sonicating on ice using an Epishear probe sonicator at a 40% power outlet (Active Motif) with two rounds of 15-sec pulses and 30-sec rest intervals. The homogenate was centrifuged at 13,000 ×g at 4°C for 15 min, and the supernatant was collected.

HT22 cells were lysed in homogenization buffer by repeated pipetting. The total protein concentrations were determined by the Bradford method (Bio-Rad Laboratories). Tissue or cell samples were mixed with 6X sample loading buffer and boiled for 5 min. Then, 20 μg of proteins per lane was resolved by SDS-PAGE, and resolved proteins were transferred onto PVDF (Bio-Rad). Transferred blots were treated with blocking solution containing 1% bovine serum albumin (BSA) or 5% skim milk in TBST (150 mM NaCl, 50 mM Tris-HCl, pH 7.4, 0.1% Tween 20). Blots were incubated with primary antibody in blocking solution at 4°C overnight. After washing three times with TBST solution for 10 min each, blots were reacted with secondary antibody dissolved in TBST solution at RT for 1 h. Immunoblots were visualized using a PicoEPD Western Reagent Kit (EBP-1073, ELPis Biotech, Daejeon, Korea). Western blot images were quantiﬁed using Image-ProPremier 6.0 software (MediaCybernetics, Rockville, MD, USA).

The primary antibodies used are: anti-AKT (9272; 1:1,000, Cell Signaling, Danvers, MA, USA), anti-amyloid beta, N-terminal specific (2C8) (M046-3; 1:1,000, Medical and Biological Laboratories, Watertown, MA, USA), purified anti-beta amyloid (6E10) (803001; 1:1,000, BioLegend, San Diego, CA, USA), anti-CaMKII (sc-13141; 1:1,000, Santa Cruz), anti-CREB1 (sc-186; 1:1,000, Santa Cruz), anti-ERK1/2 (sc-135900; 1:1,000, Santa Cruz), anti-HDAC2 (ab7029; 1:10,000, Abcam), anti-MeCP2 (3456S; 1:2,000, Cell Signaling), anti-phospho-AKT (9271; 1:500, Cell Signaling), anti-phospho-CaMKII (sc-12886-R; 1:1,000, Santa Cruz), anti-phospho-CREB (06-519; 1:1,000, Millipore), anti-phospho-ERK1/2 (4370S; 1:1,000, Cell Signaling), anti-proBDNF (ANT-006; 1:1,000, Alomone Labs, Hadassah Ein Kerem, Jerusalem, Israel), anti-REST (07-579; 1:1,000, Millipore), anti-TrkB (sc-377218, 1:500, Santa Cruz), anti-ß-actin (sc-47778; 1:1,000, Santa Cruz).

The secondary antibodies used are: anti-mouse IgG-HRP (GTX213111-01; 1:1,000, GeneTex, Irvine, CA, USA), and anti-rabbit IgG-HRP (GTX213110-01; 1:1,000, GeneTex).

**Immunocytochemistry and immunohistochemistry**

Immunocytochemical staining was carried out as described previously (Im et al., 2006). HT22 cells were plated at 2.0 × 10^4^ cells/well in a 24-well plate (SPL Life Science, Pocheon) containing a poly-D-lysine (100 μg/ml) and laminin (4 μg/ml)-coated microscope cover glass (0111520, Marienfeld Superior, Lauda-Königshofen, Germany) in DMEM containing 10% FBS and 20% PFD for 24 h. After 24 h, the culture media was changed with freshly prepared DMEM containing 1% FBS and 20% PFD, and cells were incubated for another 24 h. After washing with 1X PBS (137 mM NaCl, 2.7 mM KCl, 10 mM Na_2_HPO_4_, and 1.8 mM KH_2_PO), cells were fixed with 4% paraformaldehyde in 0.1 M phosphate buffer, pH 7.4 for 10 min at room temperature (RT) and washed with ice-cold 1X PBS. Fixed cells were permeabilized by incubating with PBS containing 0.1% Triton X-100 (PBST) for 10 min at RT and then were washed with ice-cold 1X PBS. Cells were incubated in 1% BSA dissolved in 1X PBST for 30 min to block unspecific binding and then, incubated with a primary antibody in 1% BSA solution for 1 h at RT. Cells were washed three times with 1X PBST for 5 min each and incubated with a secondary antibody in 1X PBST at RT under dark conditions. After three washes with 1X PBST, cells on the microscope cover glass (Marienfeld Superior) were mounted on the slide glass with DAPI staining mounting solution (H-1200, Vector Laboratories, Burlingame, CA, USA).

Immunohistochemical analyses were carried out as described previously (Choi et al., 2015; Kim et al., 2015). Mice were perfused with 0.9% saline via a trans-cardiac method, followed by perfusion with 4% paraformaldehyde in 0.1 M phosphate buffer, pH 7.4. After the brains were removed, they were further fixed in 4% paraformaldehyde at 4°C overnight. Vibratome-cut brain sections with a 40-μm-thickness were prepared as described above. Free-floating brain sections were incubated in 3% hydrogen peroxide dissolved in 1X PBS to block endogenous peroxidase activity at RT for 30 min and washed three times with PBST for 10 min each.

Non-specific binding was blocked with 5% BSA at RT for 1 h, and samples were incubated with a primary antibody in 5% BSA solution at 4°C overnight. Sections were washed three times with 1X PBST and incubated with a secondary antibody in 1X PBST at RT for 1 h. For immunohistochemistry, biotinylated horse anti-goat IgG (BA-9500, Vector Laboratories) and biotinylated goat anti-mouse IgG (BA-9200, Vector Laboratories) were used at a 1:200 dilution. After washing, stained signals were visualized using an ABC Elite kit (PK 6200, Vector Laboratories) and 3,3’-diaminobenzidine (D5637, Sigma-Aldrich) in 0.1 M Tris-HCl (pH 7.4). For immunofluorescent staining, a secondary antibody labeled with DyLight488 anti-rabbit (DI-1488, Vector Laboratories), DyLight594 anti-rabbit (DI-1094, Vector Laboratories), DyLight488 anti-mouse (DI-2594, Vector Laboratories), or DyLight594 anti-mouse (DI-2594, Vector Laboratories) diluted at 1:500 in 1X PBST was used. Stained sections were mounted on a gelatin-coated slide glass with fluorescent mounting medium (DAKO) and/or DAPI staining mounting solution (H-1200, Vector Laboratories).

Primary antibodies used are: anti-DCX (sc-8066, Santa Cruz), anti-HDAC2 (ab7029, Abcam), anti-Ki-67 (VP-K451, Vector Laboratories), anti-MAP2 (05-346, Millipore), anti-MeCP2 (3456S, Cell Signaling), anti-phosphoCREB (06-519, Millipore), anti-proBDNF (ANT-006, Alomone Labs), and anti-TrkB (sc-377218, 1:500, Santa Cruz),.

Histological visualization of oxidative stress levels was performed using an anti-HNE antibody, as described previously (Seo et al., 2012). 4-hydroxynonenal (HNE) is a lipid peroxidation marker, and anti-HNE antibody was purchased from Alpha Diagnostic International (HNE11-S; San Antonio, TX, USA).

Stained sections and cells were analyzed using an Olympus BX 51 microscope equipped with a DP71 camera. Stained images were analyzed using MetaMorph Microscopy Automation & Image Analysis software (Molecular device).

**Dihydroethidium (DHE) staining of brain sections**

DHE staining was carried out as described previously (Seo et al., 2012). DHE is a cell membrane-permeable, superoxide-sensitive, fluorescent dye that can be used to visualize oxidative stress levels in the brain (Seo et al., 2012). Briefly, mice were perfused with 0.9% saline via a trans-cardiac method, followed by perfusion with 4% paraformaldehyde in 0.1 M phosphate buffer, pH 7.4. After the brains were removed, they were fixed further in 4% paraformaldehyde at 4°C overnight. Vibratome-cut brain sections with a 40-μm thickness were prepared as described above. Brain sections were incubated with 1 μM DHE in 1X PBS for 5 min at room temperature under dark conditions with gentle shaking on a rotary shaker. Sections were washed three times with 1X PBS and mounted on a slide with DAPI staining mounting solution (H-1200, Vector Laboratory). DHE was purchased from Invitrogen (D1168). Fluorescence images were analyzed using a microscope equipped with a DP71 camera (BX51, Olympus) and analyzed using MetaMorph Microscopy Automation & Image Analysis software (Molecular Device).

**Malondialdehyde assay**

A malondialdehyde (MDA) assay was carried out using the Bioxytech MDA-586 kit (21044, Oxis Research, Portland, OR, USA), as described previously (Im et al., 2006; Seo et al., 2012). In brief, brain tissues were homogenized in four volumes of homogenate buffer (1X PBS containing 1% butylated hydroxytoluene) by sonication on ice with an Epishear probe sonicator (Active Motif). Homogenates were centrifuged at 3,000 g at 4°C for 10 min, and the supernatant was saved. The total protein concentration was determined by the Bradford method. Each reaction mixture consisted of 200 μl of the supernatant, 10 μl of probucol, and 640 μl of diluted R1 reagent (1:3 of methanol:Nmethyl-2-phenylindole) from the Bioxytech MDA-586 kit, and was incubated at 45°C for 60 min. The reaction was stopped by adding 150 μl of 12 N HCl, and and the mixture was centrifuged at 10,000 g for 10 min. The supernatant absorbance at 586 nm was read using the VersaMax ELISA microplate reader (Molecular Devices). MDA level was calculated with respect to the standard curve constructed with serially diluted MDA (0, 0.5, 1, 2, 3, and 4 μM). MDA data were normalized on the basis of total protein concentration and expressed as a percentage of the control value.

**MeCP2-pCREB co-immunoprecipitation assay**

Co-immunoprecipitation assays were carried out as described previously (Kim et al., 2016c), with a minor modification. The hippocampus was homogenized in 400 μl of lysis buffer (50 mM Tris–HCl, pH 8.0, 150 mM NaCl, 1% Nonidet P-40, 0.1% SDS, and 0.1% sodium deoxycholate) containing a protease inhibitor cocktail (11836153001, Roche, Indianapolis, IN, USA) by sonication on ice using an Epishear probe sonicator (Active Motif) and incubated for 30 min at 4°C. HT22 cells were plated in a 100-mm dish (1.0 × 10^6^ cells/dish) in DMEM media containing 10% FBS or 10% FBS and 20% PFD depending on the experimental design and then were incubated for 24 h. Cells were then grown in DMEM media containing 1% FBS and 20% PFD for another 24 h. Finally, 1.0 × 10^7^ cells were harvested and lysed in 400 μl of lysis buffer by sonication on ice.

Cell lysates were then centrifuged at 13,000 ×g at 4°C for 15 min, and the supernatant was collected and used for immunoprecipitation. The total protein concentration was determined using the Bradford method. One mg of proteins per each sample was pre-incubated with 10 μl of protein A/G bead (sc-2003, Santa Cruz) in 500 μl lysis buffer at 4°C for 30 min to reduce non-specific binding. The reaction was centrifuged at 13,000 ×g at 4°C for 10 min, and the supernatant was collected. The supernatant (500 μl) was added to 20 μl of protein A/G bead and 2 μg of anti-rabbit IgG (ab37415, Abcam), anti-phospho-CREB (06-519, Millipore), or anti-MeCP2 (3456S, Cell Signaling), and the reaction mixture as incubated at 4°C overnight. After centrifugation at 13,000 ×g at 4°C for 5 min, the agarose beads were saved and washed with 500 μl of washing buffer (10 mM Tris-HCl, pH 7.4, 1 mM EDTA, 1 mM EGTA, pH 8.0, 150 mM NaCl, and 0.2 mM sodium rothovanadate) containing a protease inhibitor cocktail (Roche). After centrifugation at 10,000 ×g at 4°C for 5 min, the agarose beads were washed again. The immunocomplexes bound to the agarose beads in the washing buffer were eluted by incubation with 50 μl of 0.2 M glycine, pH 2.6, for 10 min and then neutralized with 1 M Tris-HCl, pH 8.0. The eluted proteins were denatured by boiling in protein loading buffer, separated on SDS-PAGE, and processed to immunoblot analysis.

**Chromatin immunoprecipitation assay**

Chromatin immunoprecipitation (ChIP) assays were performed using the ChIP-IT® Express kit (102026, Active Motif, Carlsbad, CA, USA), as described previously (Kim et al., 2016b). In brief, mice were anesthetized using cervical translocation and the brains were surgically removed. The hippocampus was isolated and pooled for every 4-6 mice. A total of 40-50 mg of tissue samples were minced using a razor blade and immersed in 7 ml 1% formaldehyde (F1635, Sigma-Aldrich) in 1X PBS for 8 min to create crosslinks between proteins and DNA. The cross-linking reaction was stopped by adding 5 ml of 0.125M glycine solution and incubating for 5 min. After the addition of 5 ml of lysis buffer, fixed tissues were homogenized using a dounce homogenizer with the “A” pestle (PYREX® Dounce Tissue Grinders). Then, homogenates were centrifuged at 1,250 ×g at 4°C for 10 min, and the pellet containing the nuclei was collected, resuspended in 500 μl of lysis buffer containing a proteinase cocktail (0.5X final) and PMSF (0.5 mM final), incubated on ice for 30 min, and homogenized using a dounce homogenizer with the “B” pestle.

HT22 cells grown to 3.0 × 10^7^ cells on a 100-mm dish were used for drug treatment. After washing with 1X PBS (137 mM NaCl, 2.7 mM KCl, 10 mM Na_2_HPO_4_, 1.8 mM KH_2_PO_4_), cells were treated with 1% formaldehyde in 1X PBS to cross-link proteins and DNA at room temperature for 10 min. The fixation was stopped by adding 5 ml of 0.125M glycine and incubating for 5 min. After washing with 1X PBS, cells were harvested in 5 ml of 1X PBS containing proteinase cocktail (0.5X final) and PMSF (0.5 mM final), and centrifuged at 1,250 ×g at 4°C for 10 min. The cell pellet was resuspended in 500 μl of lysis buffer containing a proteinase cocktail and PMSF, incubated on ice for 30 min, and homogenized using a dounce homogenizer with the “B” pestle.

The homogenates were centrifuged at 1,250 ×g at 4°C for 10 min, and then the pellet containing fixed chromatins was resuspended in 300 μl of shearing buffer containing proteinase cocktail and PMSF and placed on ice. The fixed chromatins in the buffer were sheared to 200-800 bp sizes by sonication on ice with 20-sec pulses and 50-sec rest intervals, repeated 20 times for tissue samples or 10 times for HT22 cell samples, using an Epishear probe sonicator set into a 35% power outlet (Active Motif, Carlsbad, CA, USA). After centrifugation at 22,000 ×g at 4°C for 10 min, the supernatant containing sheared chromatin was saved, and the DNA content was quantified using a NanoDrop machine (Thermo Fisher Scientific, Wilmington, DE, USA). Proper chromatin shearing was confirmed by agarose gel electrophoresis and sheared chromatin samples were used immediately or stored at -80°C.

For quantification, 10 μl of sheared chromatin samples was set aside to use as an input DNA control. For immunoprecipitation, 10 μg of sheared chromatin was added to 2 μg of primary antibody, and 20 μl of protein G magnetic beads in 100 μl of 1X ChIP Buffer I containing a proteinase inhibitor cocktail (1X final) and rocked gently at 4°C overnight. The antibodies used were anti-MeCP2 (3456S; Cell Signaling), anti-phospho-CREB (06-519; Millipore), and non-immune rabbit IgG (ab37415; Abcam) as an antibody binding control. By standing sample tubes on a magnetic rack, the magnetic beads containing immunoprecipitates were washed with 200 μl of ChIP buffer I, followed by 200 μl of ChIP buffer II twice. After the final wash, the beads were resuspended in 50 μl of elution buffer and incubated for 15 min at room temperature with gentle rocking. After adding 50 μl of reverse cross-linking buffer, the supernatant containing eluted chromatins was collected and incubated at 95°C for 15 min. The sheared chromatins (10 μl) that were saved to use as input DNA control were mixed with 88 μl of ChIP buffer II and 2 μl of 5 M NaCl and were incubated at 95°C for 15 min. The reaction mixtures were then added to 1 μg of Proteinase K, incubated at 37°C for 1 h, and then mixed with 2 μl of Proteinase K stop solution. The final reaction mixture (100 μl) was regarded as immunoprecipitated DNA and used for real-time PCR.

Immunoprecipitated DNA was used for quantitative real-time PCR, as described above. One-tenth of the input DNA was used for quantitative PCR to control for the relative amount of DNA fragments in immunoprecipitation. Immunoprecipitated quantities were calculated by following the method, as described previously (Kim et al., 2015). ChIP-qPCR data were analyzed by normalization of the ChIP signal relative to that of the input DNA using the following formulas.

$$100*2^[Ct \left( adjusted input \right)-Ct \left( \mathrm{ChIP} \right)]$$

The Ct values were computed by the CFX 96 Real-Time PCR System Detector (Bio-Rad Laboratories). Samples used for ChIP assays were obtained from more than two independent sets and quantitative PCR was repeated more than three times.

The primers were designed using the Ensemble Genome Browser (<http://asia.ensembl.org/index.html>), Primer3Web version 4.1.0 (<http://primer3.ut.ee/>), and the analyzed primer pairs were designed using an *in silico* PCR program (the USCS Genome Browser at <http://genome.cse.ucsc.edu/cgi-bin/hgPcr>). The primers used are; the *Arc* promoter, forward 5’-CAGCATAAATAGCCGCTGGT-3’ and reverse 5’-AGTGTGGCAGGCTCGTC-3’; *Bdnf1* promoter, forward 5’- TGATCATCACTCACGACCACG-3’ and reverse 5’-CAGCCTCTCTGAGCCAGTTACG-3’; *Bdnf3* promoter, forward 5’-GTGAGAACCTGGGGCAAATC-3’ and reverse 5’-ACGGAAAAGAGGGAGGGAAA-3’; *Bdnf4* promoter, forward 5’-CTTCTGTGTGCGTGAATTTGCT-3’ and reverse 5’-AGTCCACGAGAGGGCTCCA-3’; *Grin1* promoter, forward 5’- TCCTATCTCACCCTCTAGAGTGT-3’ and reverse 5’- TGTGAATGCTTGAGTGTGCG-3’; *Nt3* promoter, forward 5’-CAATGCACTCTTCCTGACGT-3’ and reverse 5’-GGAGGGAGGAGGGGTCTAC-3’; *Grin2b*, forward 5’-CGGGTTAAGCTTTTCCCACC-3’ and reverser 5’-GTCCTCTTGCTCTCCACCC-3’; *Nt4/5* promoter, forward 5’-AACTTGTGGCCATCTTCCTG-3’ and reverse 5’-GTGGCAGACTATGGGAGGAC-3’.

**Stereotaxic injection of siRNA**

Stereotaxic injection of siRNA was performed as described previously (Choi et al., 2015; Kim et al., 2015). In brief, mice were anesthetized with a 3.5 : 1 mixture of ketamine hydrochloride (50 mg/ml) and xylazine hydrochloride (23.3 mg/ml) at a dose of 2.5 μl/g body weight. Control siRNA (siCON, SN-1012), MeCP2-siRNA (siMeCP2, 1385135; NM_001081979.2), TrkB-siRNA (siTrkB, 1393919; NM_008745.3), CREB-siRNA(siCREB, 1342686; NM_009952.2), and BDNF-siRNA (siBDNF, 1330617; NM_007540.4) were purchased from Bioneer Co. (Daejeon, Korea). The siRNAs were resolved to 50 ng/μl in siRNA dilution buffer (B-002000-UB-100, Dharmacon, Lafayette, CO, USA).

One volume of diluted siRNA (50 ng/μl) was mixed with 2.5 volumes of Neurofect transfection reagent (T800075, Genlantis, San Diego, CA, USA) and 0.5 volumes of 50% sucrose and incubated on ice for 20 min prior to injection into the brain. The siRNA mix (1.8 μl of 7.5 ng/μl) was injected into each CA3 (stereotaxic coordinate: AP, -1.9; ML, ±3.0; DV, -2.1 mm) at the speed of 0.2 μl/min using a stereotaxic injection system (Vernier Stereotaxic Instrument, Leica Biosystems, Wetzlar, Germany), and a Hamilton syringe with a 30-G needle. After 5 min, the needle was removed in three intermediate steps of 3 min each to minimize backflow, and mice were kept on a warm pad until they were awakened. The novel object recognition test and novel location recognition test was performed between 48 h and 72 h after injection of the siRNA.

**Transfection of siRNA into HT22 cells**

Transfection of siRNA into HT22 cells was carried out using Lipofectamine-2000 (13778-075; Invitrogen), as described previously (Han et al., 2013; Lee and Jeong. 2016). HT22 cells were plated at a concentration of 8.0 × 10^4^ cells/well in a 6-well plate (SPL Life Science, Pocheon-si) and grown in DMEM containing 10% FBS with or without PFD. After 24 h, the media containing DMEM containing 1% FBS with or without PFD was changed, and transfection with siRNA was carried out as below. Lipofectaime-2000 (9 μl) and 20 μM siRNA (3 μl) were separately diluted in 150 μl of Opti-MEM^®^ Medium (31985070, Gibco, Thermo Fisher Scientific, Paisley, Scotland, UK). Diluted siRNA was mixed with diluted Lipofectamine-2000 at 1:1 ratio, and the mixture was incubated for 5 min at room temperature. Then, the siRNA-Lipid complex (250 μl) was gently dripped onto HT22 cells in 6-well plates and cells were grown for 24 h. The final concentration of siRNA was 50 pM and 7.5 μl/well of Lipofectamine-2000 was used.

**Behavioral tests**

Behavioral tests were carried out as described previously (Lee et al., 2006; Choi et al., 2015). Behavioral performance was recorded with a computerized video-tracking system (SMART; Panlab S.I., Barcelona, Spain) and a webcam recording system (HD Webcam C210, Logitech, Newark, CA, USA). Mice were habituated for 30 min at the behavior testing room prior to starting each behavioral test. The background sound in the testing room was masked with 65 dB of white noise. All behavioral tests were performed during the light cycle (9 a.m. – 3 p.m.). After each behavioral test, all parts of the apparatus that had been exposed to mice were cleaned with 70% ethanol. The behavior testing room was lit up to 20 lux with two indirect lighting sources.

***Water maze test***

The water maze test was performed as described previously (Lee et al., 2006; Choi et al., 2015). Briefly, the training was performed in a circular tank pool (diameter, 90-cm) filled with opaque water (made using Sargent^®^ White Art Tempera Paint) and placed in the center of a room with environmental cues on each side of the quadrants. The water temperature was kept between 23-24°C. The platform (diameter, 10 cm) was submerged 2 cm below the water surface of a quadrant, which was called the target quadrant.

On a daily training schedule, mice were placed into the maze at a random point and allowed to search for the platform until reaching the platform or for a maximum of 90 sec. The latency time (sec) to reaching the platform was recorded. If the mice did not find the platform in 90 sec, they were guided gently to stand on it for 30 sec. The training was performed twice a day with a 6-h interval between trials for five days.

On day 6, mice were given the probe trial test in which they were allowed to explore the maze pool with no platform for 60 sec. The swimming path of each mouse was analyzed. The periphery was defined as the area between the wall of the swimming pool tank and the circular line running 10 cm from the wall. At the end of the experiment, mice were given the visible platform test to control for possible locomotor and visual deficits, in which they were allowed to explore the maze with the visible platform present, created by placing a 7-cm vertical pole with a triangle black flag (base, 4.5 cm and height, 4.5 cm) on top of the submerged platform.

***Novel object recognition tests***

The standard and modified novel objective recognition tests were performed as described previously (Kim et al., 2016a) with a minor modification. The standard novel objective recognition test was performed in an open field (40 cm x 30 cm) in a dimly lit (20 lux) behavioral testing room. First, a subject mouse was presented to two identical objects (object A, wooden blocks; 3.5 cm x 3.5 cm x 7 cm), which were placed 20 cm apart in the open field, and was allowed to familiarize itself with the two objects for 10 min. The amount of time the mouse spent exploring each object with its nose or approaching an object (within 2 cm) was recorded, whereas wandering around the object was not considered an exploration. This familiarization step was repeated three times with 10-min intervals between each session.

Two hours after the familiarization sessions, the familiarized objects were replaced with one of the familiar objects (object A) and a new object (object B, a 100-ml glass flask containing fresh cage bedding at a 3-cm depth; 6 cm in diameter x 10 cm in height). The amount of time spent exploring each object was recorded for 10 min. There was no bias observed in the reverse trial in which two 100-ml glass flasks were used as the familiar objects and a wooden block was used as the novel object. Twenty-four hours after the familiarization sessions, the subject mouse was presented to a familiar object (object A) and a third, new object (object C, a plastic block made by stacking four 60-mm culture dishes with a black-tape banding; 5.5 cm in diameter x 7.5 cm in height) replacing the previous novel object (the 100-ml glass flask containing cage bedding), and the time spent with each object was recorded for 10 min, and analyzed in a blind manner with two researchers.

***Modified novel object recognition test and novel location recognition test***

A series of spatial cue-associated modified novel object recognition tests were carried out to examine whether siRNA-mediated knockdown of a specific gene in the hippocampus changed the effects of HO_2_ on improving memory retention.

A spatial cue was provided on top of the wall directly outside of the open field apparatus by posting a black circle (26-cm outer diameter and 19-cm inner diameter) to help the animal orient itself spatially. The experiment consisted of a series of novel object recognition and spatial relocation recognition memory tests.

Two identical objects (object A; wooden blocks; 3.5 cm x 3.5 cm x 7 cm) were placed 30 cm apart in a diagonal direction in the open field, so that one was located close to the black circle side, while the other was on the opposite side. First, a subject mouse was allowed to explore the open field with no objects to habituate for 5 min. Then, a subject mouse was familiarized with two identical objects (object A) for 10 min, the amount of time spent exploring each object was recorded, and this familiarization process was repeated.

Two hours later, the subject mouse was placed in the open field in which the object close to the black circle marker was replaced with a new object (object B, a 100-ml glass flask containing cage bedding, as above). The amount of time spent exploring each object was recorded for 10 min (NOR test).

After 15 min of the NOR test, the subject mouse was presented in the open field in which the old familiar object A was moved to a novel location toward the black circle and 20 cm away from object B. The amount of time spent exploring each object was recorded for 10 min (NLR test), and analyzed in a blind manner with two researchers.

***Passive avoidance test***

The passive avoidance test has been described previously (Lee et al., 2006; Kim et al, 2012). The test apparatus consisted of two spaces, an overhead lit bright chamber and a dark chamber equipped with a metal grid floor and a door between the two chambers (each chamber, 15 x 15 x 15 cm). On the first day, subject mice were individually placed in the lighted chamber (1,500 lux) with the door opened and allowed to explore freely the equipment for 5 min. On the second day, mice were placed in the lighted chamber with the door closed and allowed to explore for 30 sec. Then, the door was opened and the latency to entering the dark chamber with all four paws was recorded as the pre-shock value. When a mouse entered the dark chamber completely, the door was closed and two foot-shocks (100 V, 0.3 mA, 2-s shock duration with a 5-sec interval) were delivered through the grid floor. After 30 s, the mouse was returned to its home cage. On the test day, 24, 72, and 120 h after training, the mouse was placed in the lighted chamber with the door open, and the latency to entering the dark chamber, with a cut-off time of 300 s, was recorded and regarded as the post-shock value. The total freezing time during the testing period was manually analyzed..

**Statistical analysis**

Two-sample comparisons were carried out using Student’s *t*-test, whereas multiple comparisons were performed using one-way ANOVA followed by the Newman-Keuls *post hoc* test or two-way ANOVA or two-way repeated measures ANOVA followed by the Bonfferoni *post hoc* test. GraphPad PRISM 6.0 software (GraphPad Software. Inc., La Jolla, CA, USA) was used for statistical analyses. All data are presented as mean ± SEM, and statistical significance was accepted at the 5% level.

Han HE, Kim TK, Son HJ, Park WJ, Han PL. Activation of Autophagy Pathway Suppresses the Expression of iNOS, IL6 and Cell Death of LPS-Stimulated Microglia Cells. Biomol Ther (Seoul) 2013; 21: 21-8.

Im JY, Kim D, Paik SG, Han PL. Cyclooxygenase-2-dependent neuronal death proceeds via superoxide anion generation. Free Radic Biol Med 2006; 41:960–72.

Jankowsky JL, Slunt HH, Ratovitski T, Jenkins NA, Copeland NG, Borchelt DR. Co-expression of multiple transgenes in mouse CNS: a comparison of strategies. Biomol Eng 2001; 17: 157-65.

Kim C, Yun N, Lee J, Youdim MB, Ju C, Kim WK, et al. Phosphorylation of CHIP at Ser20 by Cdk5 promotes tAIF-mediated neuronal death. Cell Death Differ 2016c; 23: 333-46.

Kim TK, Kim JE, Park JY, Lee JE, Choi J, Kim H, et al. Antidepressant effects of exercise are produced via suppression of hypocretin/orexin and melanin-concentrating hormone in the basolateral amygdala. Neurobiol Dis 2015; 79: 59-69.

Lee DS, Jeong GS. Butein provides neuroprotective and anti-neuroinflammatory effects through Nrf2/ARE-dependent haem oxygenase 1 expression by activating the PI3K/Akt pathway. Br J Pharmacol 2016; 173: 2894-909.

Lee KW, Im JY, Song JS, Lee SH, Lee HJ, Ha HY, et al. Progressive neuronal loss and behavioral impairments of transgenic C57BL/6 inbred mice expressing the carboxy terminus of amyloid precursor protein. Neurobiol Dis 2006; 22: 10-24.

Savonenko A, Xu GM, Melnikova T, Morton JL, Gonzales V, Wong MP, et al. Episodic-like memory deficits in the APPswe/PS1dE9 mouse model of Alzheimer’s disease: relationships to beta-amyloid deposition and neurotransmitter abnormalities. Neurobiol Dis 2005; 18: 602-17.

Seo JS, Kim TK, Leem YH, Lee KW, Park SK, Baek IS, et al. SK-PC-B70M confers anti-oxidant activity and reduces Abeta levels in the brain of Tg2576 mice. Brain Res 2009; 1261: 100-8.

Seo JS, Lee KW, Kim TK, Baek IS, Im JY, Han PL. Behavioral stress causes mitochondrial dysfunction via ABAD up-regulation and aggravates plaque pathology in the brain of a mouse model of Alzheimer disease. Free Radic Biol Med 2011; 50: 1526-35.

Seo JS, Park JY, Choi J, Kim TK, Shin JH, Lee JK, et al. NADPH oxidase mediates depressive behavior induced by chronic stress in mice. J Neurosci 2012; 32: 9690-9.
